# Chemical proteomics reveals the target landscape of 1,000 kinase inhibitors

**DOI:** 10.1038/s41589-023-01459-3

**Published:** 2023-10-30

**Authors:** Maria Reinecke, Paul Brear, Larsen Vornholz, Benedict-Tilmann Berger, Florian Seefried, Stephanie Wilhelm, Patroklos Samaras, Laszlo Gyenis, David William Litchfield, Guillaume Médard, Susanne Müller, Jürgen Ruland, Marko Hyvönen, Mathias Wilhelm, Bernhard Kuster

**Affiliations:** 1 Chair of Proteomics and Bioanalytics, Technical University of Munich, Freising, Germany; 2 German Cancer Consortium (DKTK), partner site Munich and German Cancer Research Center (DKFZ), Heidelberg, Germany; 3 Department of Biochemistry, University of Cambridge, Cambridge, UK; 4 Institute of Clinical Chemistry and Pathobiochemistry, School of Medicine, Technical University of Munich, Munich, Germany; 5 Center for Translational Cancer Research (TranslaTUM), Munich, Germany; 6 Structural Genomics Consortium, Buchmann Institute for Life Sciences, Goethe University Frankfurt, Frankfurt, Germany; 7 Institute of Pharmaceutical Chemistry, Goethe University Frankfurt, Frankfurt, Germany; 8 Department of Biochemistry, Schulich School of Medicine and Dentistry, Western University, London, Ontario Canada; 9 German Center for Infection Research (DZIF), partner site Munich, Munich, Germany; 10 Computational Mass Spectrometry, Technical University of Munich, Freising, Germany; 11 Bavarian Biomolecular Mass Spectrometry Center (BayBioMS), Technical University of Munich, Freising, Germany

**Keywords:** Proteomics, Kinases, Small molecules, Target identification

## Abstract

Medicinal chemistry has discovered thousands of potent protein and lipid kinase inhibitors. These may be developed into therapeutic drugs or chemical probes to study kinase biology. Because of polypharmacology, a large part of the human kinome currently lacks selective chemical probes. To discover such probes, we profiled 1,183 compounds from drug discovery projects in lysates of cancer cell lines using Kinobeads. The resulting 500,000 compound–target interactions are available in ProteomicsDB and we exemplify how this molecular resource may be used. For instance, the data revealed several hundred reasonably selective compounds for 72 kinases. Cellular assays validated GSK986310C as a candidate SYK (spleen tyrosine kinase) probe and X-ray crystallography uncovered the structural basis for the observed selectivity of the CK2 inhibitor GW869516X. Compounds targeting PKN3 were discovered and phosphoproteomics identified substrates that indicate target engagement in cells. We anticipate that this molecular resource will aid research in drug discovery and chemical biology.

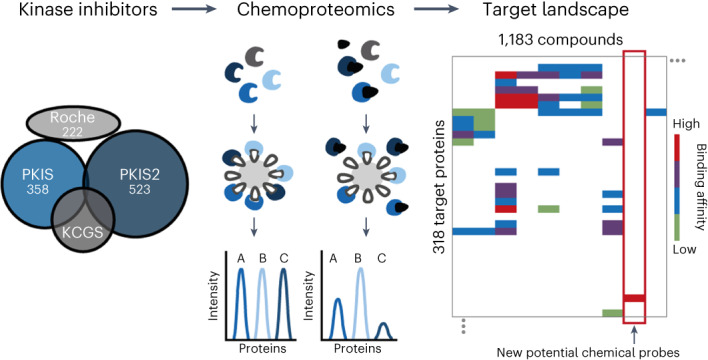

## Abstract (web summary)

Chemical proteomics profiling of 1,183 kinase inhibitors from past drug discovery projects revealed a large number of reasonably selective compounds for several kinases such as SYK and CK2.

## Main

Kinase inhibitors have become important drugs, particularly in oncology. About 80 have been approved for use in humans and hundreds are investigated in clinical trials^1^. Most of these drugs show substantial polypharmacology, which may enhance their efficacy but may also result in undesired off-target liabilities^2,3^. Kinase inhibitors also play a major role as chemical probes in basic research, for example to explore the function of a particular kinase in a defined biological context or to validate it as a therapeutic target. To be able to attribute observed phenotypic or molecular effects of a compound to the inhibition of a particular kinase target, chemical probes must meet a series of stringent criteria including high potency, cellular activity and selectivity^4–6^.

Despite tremendous efforts in medicinal chemistry, many kinases still lack highly selective inhibitors and there are substantial errors in the literature regarding conclusions drawn from experiments using unselective compounds^7,8^. One way to overcome these issues is to use tool compounds with known selectivity profiles and broad annotation in several different assay panels^9^. To promote the development of such chemical probes and to foster research on kinases that have received little attention thus far, collections of well-characterized compounds have been assembled. This includes two versions of the published kinase inhibitor set (PKIS and PKIS2) from drug discovery programs of GlaxoSmithKline, Pfizer and Takeda^10,11^. Both sets have been widely distributed in the research community to crowd source assays that further characterize these molecules, to identify chemical starting points for the development of new chemical probes or to investigate kinase signaling. Because PKIS and PKIS2 still contain compounds that are too promiscuous or of insufficient potency to qualify as chemical probes, industrial and academic partners have teamed up to create the Kinase Chemogenomic Set (KCGS) comprising 187 small molecule kinase inhibitors^12^. All KCGS compounds show potent kinase inhibition and high selectivity when screened across a large panel of biochemical assays. A conceptually similar library has been assembled from peer-reviewed publications of projects performed at Hoffmann-La Roche Inc. However, none of the above compound sets has been investigated for selectivity on a proteome-wide scale.

We and others have shown that chemical proteomics approaches using immobilized kinase inhibitors (Kinobeads^13,14^, MIBs^15^) or biotinylated acylphosphate probes (KiNativ^16^) as affinity tools are an efficient and quantitative means to explain an inhibitors’ target binding and selectivity profile under close-to-physiological conditions. Hence, the aim of the current study was (1) to characterize the target space and selectivity of the 1,183 published tool compounds assembled in the PKIS, PKIS2, KCGS and Roche collections, (2) to exemplify how these data may be used to identify potential new chemical probes, (3) to shed light on their mechanisms of action and (4) to share this resource of drug–target interaction data with the scientific community to aid in drug discovery as well as chemical probe design for understudied kinases.

## Results

### The target landscape of 1,183 tool compounds

**Fig. 1:**
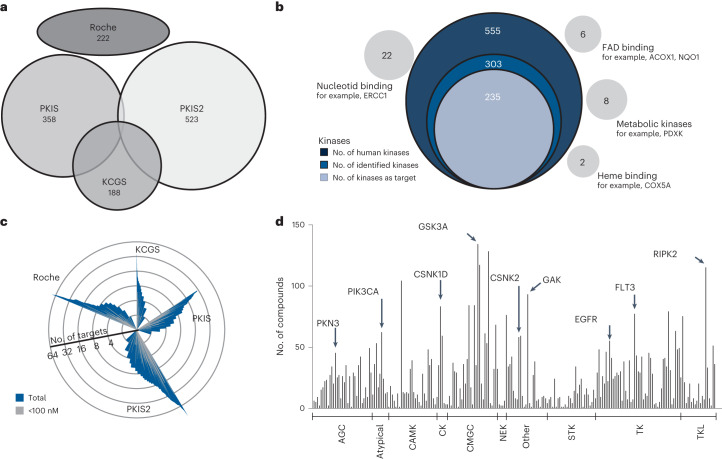
The target landscape of 1,184 kinase tool compounds. **a**, Small molecule kinase inhibitor libraries (including number of compounds in each library) used for Kinobeads profiling. **b**, Venn diagram of the number of kinases in the human genome, enriched by Kinobeads from mixed lysates and targeted by at least one inhibitor. Several metabolic kinases as well as nucleotide, FAD and heme binding proteins were also identified as kinase inhibitor binders. **c**, Windmill plot grouping compounds by library and the number of targets by Kinobeads. Compounds with potency below 100 nM are marked in gray. **d**, Number of compounds targeting a specific kinase with an affinity below 1 µM. Kinases are grouped by subfamilies and sorted alphabetically within each group.

**Extended Data Fig. 1:**
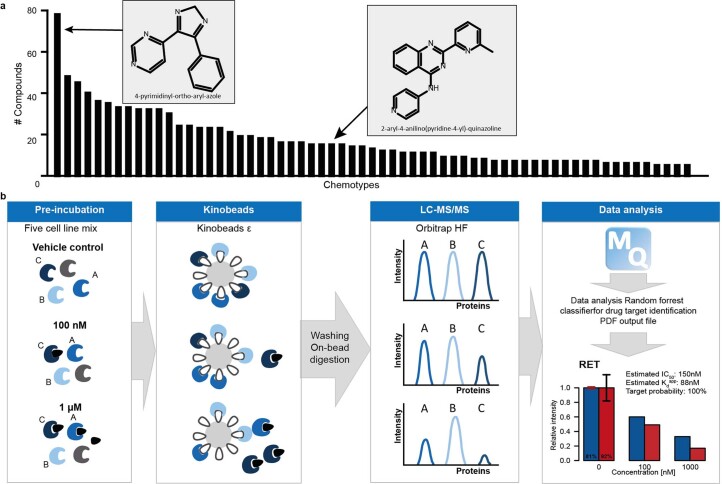
Screening setup. **a**, Tool compounds were grouped into different chemotypes. The height of bars represents the number of compounds per chemotype. Representative structures of two chemotypes are given. Compound grouping was based on literature data. **b**, Schematic representation of the Kinobeads workflow used to profile drug-protein interactions. Cell lysates were equilibrated with DMSO, 100 nM or 1 µM of each drug. Kinobeads ε were used to enrich kinases and other proteins from lysates. Bead bound proteins were digested on-beads and peptides were measured by liquid chromatography-tandem MS. An automatic data analysis pipeline including a random forest classifier was used for peptide and protein identification and quantification, data processing and target annotation.

The four kinase inhibitor sets characterized here (PKIS, PKIS2, KCGS, Roche) comprise 1,183 nonredundant (111 duplicates) small molecules with drug-like physicochemical properties representing 64 chemotypes with high structural diversity (Fig. 1a, Extended Data Fig. 1a and Supplementary Table 1). All compounds were subjected to Kinobead competition binding profiling at two concentrations (100 nM and 1 µM) and using a mixture of lysates from five cancer cell lines (K-562, COLO-205, MV-4-11, SK-N-BE(2) and OVCAR-8) to maximize the representation of endogenous proteins (Extended Data Fig. 1b, see ‘Kinase inhibitor profiling with Kinobeads (Kinobeads pulldowns)’ in Methods section for details). Briefly, Kinobeads comprise seven broad-spectrum small molecule kinase inhibitors immobilized on Sepharose beads. This enables affinity enrichment of about 300 of the 555 human protein and lipid kinases as well as hundreds of further proteins from native cell lysates^13,14,17^ (Fig. 1b). Compounds of interest are set to compete for target protein binding with Kinobeads in the lysate and the amount of a given protein bound to Kinobeads in the presence of a compound can be quantified relative to a dimethylsulfoxide (DMSO) vehicle control by label-free mass spectrometry. This assay measures the physical interaction of a compound with thousands of endogenous proteins in parallel and, when systematically increasing the concentration of competitor, enables the calculation of an apparent interaction constant (${{{K}}}_{{\rm{d}}}^{{\rm{app}}}$) for each compound and protein.

**Extended Data Fig. 2:**
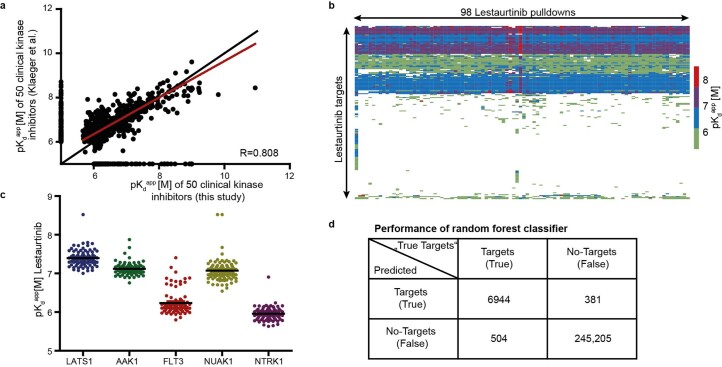
Screening controls. **a**, p*K*_d_^app^ values of targets of 50 clinical kinase inhibitors obtained with two doses were correlated to previously published results of full dose response (8 doses) Kinobeads experiments (Klaeger et al.^3^). Each dot represents one drug-protein combination. Black line indicates a perfect correlation of 1. **b**, Unsupervised clustering of 98 Lestaurtinib pulldowns and their targets (color code reflects the p*K*_d_^app^ of the drug protein interaction). **c**, Distribution of p*K*_d_^app^ values for one of the indicated targets over 98 pulldown experiments. **d**, Performance of the random forest classifier used for target annotation was determined based on 98 Lestaurtinib Kinobeads pulldown experiments. Lestaurtinib has 76 targets according to Kinobeads screening data and numbers of false positives and false negatives were calculated.

To enhance throughput, we investigated if two compound competitor concentrations (100 nM and 1 µM) are sufficient to determine ${{{K}}}_{{\rm{d}}}^{{\rm{app}}}$ values. Therefore, 50 clinical kinase inhibitors were profiled with two inhibitor concentrations and the results were compared to full dose–response data previously published for the same compounds^3^ (Extended Data Fig. 2a and Supplementary Tables 1 and 2). We found good overall agreement between the two assays (Pearson’s *r* = 0.808) but note that ${{{K}}}_{{\rm{d}}}^{{\rm{app}}}$ values obtained from the two-dose data are only rough approximations, particularly for weak compound–protein interactions and should, therefore, be treated with caution.

Further adjustments to the published protocol included the reduction of the amount of protein extract per experiment to 2.5 mg of protein and to 17 µl of settled Kinobeads to enable higher throughput. Six DMSO vehicle controls were included in random order on every 96-well plate to allow data normalization within and across plates. We developed a data analysis pipeline including protein identification and quantification by MaxQuant/Andromeda^18^, calculation of half-maximum inhibitory concentration (IC_50_) and ${{{K}}}_{{\rm{d}}}^{{\rm{app}}}$ values, creation of interaction plots and classification of proteins as targets (see Methods section ‘Data analysis of Kinobeads pulldowns for details’)^19^. Briefly, we used a random forest classifier for target annotation that was trained on residual binding of proteins to beads, the number of peptides identified for a given protein, the number tandem mass spectra that gave rise to these identifications and intensity variations within the DMSO controls.

To assess the reproducibility of the assay, triplicates of the tyrosine kinase inhibitor lestaurtinib were included in each 96-well plate. Lestaurtinib was chosen because it has 76 targets in the Kinobead assay that span a large range of affinities. Most targets were identified with similar affinities in each of the 98 lestaurtinib experiments indicating good intraplate and interplate reproducibility of the assay (Extended Data Fig. 2b,c). More specifically, we measured a false positive rate of 0.16% and a false negative rate of 6.8% leading to an overall assay performance of 93.2% sensitivity and 99.8% specificity (Extended Data Fig. 2d).

**Extended Data Fig. 3:**
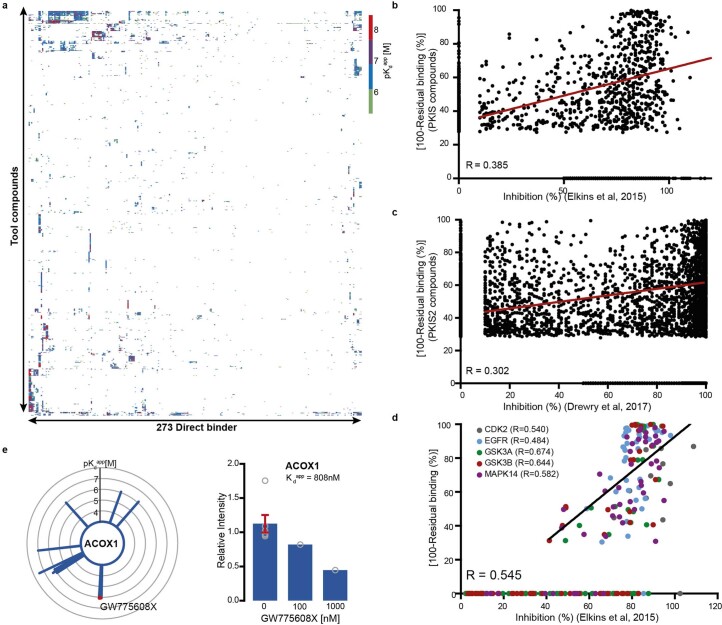
The target landscape of 1,184 tool compounds. **a**, Hierarchical clustering of Kinobeads direct binders against 1,184 tool compounds. Each square represents one compound-target interaction and the color reflects the affinity of the interaction. **b**, Correlation of inhibition data of PKIS derived from Elkins et al.^10^ against binding data of the Kinobeads screen. Each dot represents one drug-target pair. Dots on x- and y- axis were only identified as targets in one of the screens. **c**, Correlation of inhibition data of PKIS2 derived from Drewry et al.^11^ to the binding data of the Kinobeads screen. Each dot represents one drug-target pair. Dots on x- and y- axis were only identified as targets in one of the screens. **d**, Correlation of inhibition data of PKIS derived from Elkins et al.^10^ against binding data of the Kinobeads screen only for the cognate target of designated CDK2, EGFR, GSK and MAPK14 inhibitors. Each dot represents one drug-target pair. Dots on x- and y- axis were only identified as targets in one of the screens. Goodness of fit (R values) for each compound group as well as the overall correlation are displayed. **e**, Radar plot displays compounds that bind to ACOX1 (each spike is a drug and the length correspond to the affinity of the interaction). Right panel shows dose dependent intensity reduction of ACOX1 with increasing concentrations of GW775608X. Mean values +/−s.e.m. of *n* = 6 DMSO control pulldown experiments are shown.

A total of 235 kinases were targeted by at least one inhibitor (Fig. 1b) and 226 kinases showed submicromolar affinity for at least one compound. The number of targets per compound varied greatly between compounds (one to more than 100) and this was observed for all four libraries (Fig. 1c). No targets could be identified for 67 compounds and a further 194 compounds had no target with submicromolar affinity. Only compound–target interactions with nanomolar affinity were considered for all further analysis because the affinity data are not reliable beyond the highest dose in the assay (1,000 nM). Hierarchical clustering of compounds (*n* = 833) and their respective protein kinase targets (*n* = 226) revealed 5,341 nanomolar interactions illustrating that about half of the kinome can be drugged by the compound set analyzed here (Extended Data Fig. 3a and Supplementary Table 2).

We found a poor overall correlation (Pearson’s *r* = 0.385 and *r* = 0.302) between the two-dose affinity data from Kinobeads and published single-dose enzymatic inhibition data using recombinant kinase assays (Nanosyn and KINOMEscan) for PKIS and PKIS2 (refs. ^10,11^) (Extended Data Fig. 3b,c). The correlations were often better when considering the designated targets of the tool compounds only (Pearson’s *r* = 0.484–0.674; Extended Data Fig. 3d). The reasons for such apparent discrepancies between assays have been discussed before and can be explained by the multitude of different assay conditions including ATP concentrations, activities of native versus recombinant proteins, presence versus absence of cellular cofactors and complex partners or posttranslational modification of kinases and so on, all of which may affect binding and/or activity^3,20^ (see ‘Discussion’).

The four compound sets targeted a broad range of kinases from all subfamilies with a slight overrepresentation of tyrosine kinases and CMGC kinases (Fig. 1d). This may be rooted in the fact that early kinase drug discovery often focused on the same few kinases. The most frequently targeted kinases were GSK3A, MAPK14, GAK, RIPK2 and RET with more than 100 compounds each. GAK and RET were particularly frequently hit even though very few compounds of the libraries analyzed were specifically designed to target either of the two proteins. Very similar observations have been made using Nanosyn and KINOMEscan assays^10,11^. In addition to kinases, Kinobeads also bind hundreds of other proteins, offering the possibility to discover unexpected interactions. Specific such interactions were identified for 16 nucleotide binding (nonkinase) proteins, five flavin adenine dinucleotide (FAD) binding proteins, two heme binding proteins and four metabolic kinases (Fig. 1b). Examples include the known off-targets NQO2 (ref. ^21^) and FECH^22^, and novel cases such as the FAD binding proteins ACOX1 and NQO1 (Extended Data Fig. 3e).

### Selectivity of kinase inhibitor tool compounds

**Fig. 2:**
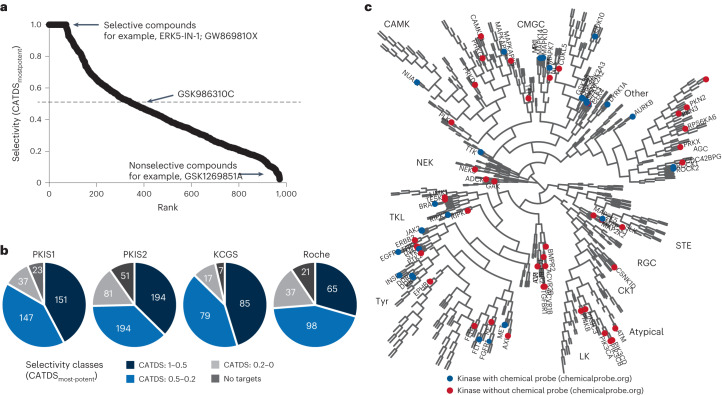
Selectivity of kinase tool compounds and potential chemical probes. **a**, Selectivity ranking of tool compounds (according to the compound-centric selectivity score, CATDS_mostpotent_) highlighting ERK5-IN-1 and GW869810X as selective and GSK1269851A as unselective inhibitors. **b**, Distribution of the number of tool compounds over categories of selectivity. **c**, Phylogenetic tree of kinases marking kinases for which chemical probes have been identified in this study (blue and red). Blue circles represent kinases for which chemical probes have been reported before.

**Extended Data Fig. 4:**
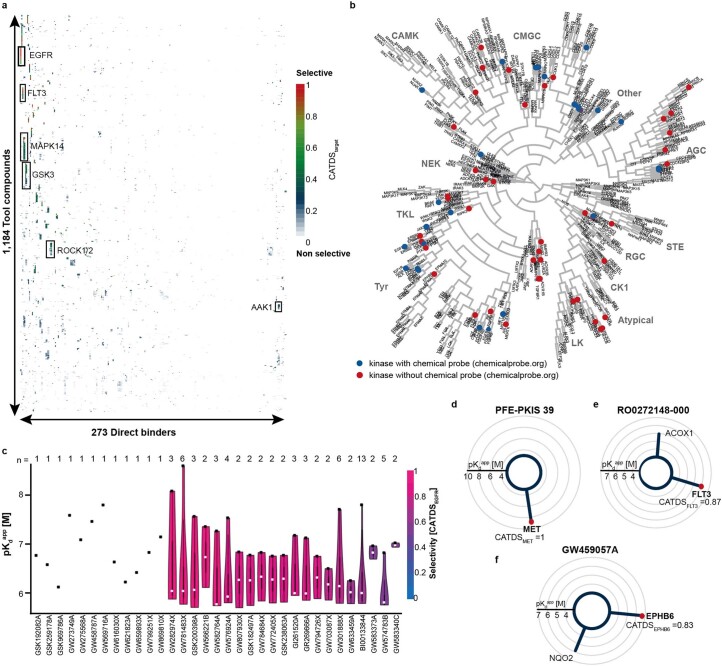
Identification of potential chemical probes. **a**, The CATDS_target_ score of all 1,184 tool compounds and their corresponding direct binder targets are assembled in a drug/target interaction matrix using unsupervised clustering. Several clusters are highlighted in boxes. **b**, High resolution phylogenetic tree of Fig. 2 with labels of all kinases. Blue circles represent kinases for which chemical probes have been reported before. **c**, Violin plots comparing the potency and selectivity of EGFR inhibitors that were identified as chemical probes in this study. p*K*_d_^app^ values of EGFR are marked as black dots. The shape of the violin indicates the number of targets at the respective p*K*_d_^app^ and the color reflexed the selectivity for EGFR. The total number of targets is printed at the top. The white dots represent the median of the data, the black boxes represent the first and third quartiles. Violins are colored according to selectivity for EGFR (CATDS_EGFR_). **d-f**, Radar plots depicting proteins that bind to PFE-PKIS39 **d**, RO0272148-000 **e** or GW459057A **f**. Each spike is one protein target and the length corresponds to the affinity of the interaction. Source data

Selectivity is a major characteristic for the designation of a kinase inhibitor as a chemical probe and we have introduced a selectivity metric called CATDS (concentration and target dependent selectivity) that provides a more accurate measure of selectivity than merely counting the number of targets at a particular drug concentration^3,23^. We note that the use of the term ‘selectivity’ throughout this manuscript solely refers to proteins that can be assayed by Kinobeads. CATDS_most potent_ equates to the half-maximal engagement of the most potent compound–target interaction divided by the sum of all target engagements of the compound at that concentration. CATDS scores near one indicate very selective compounds regardless of which protein they target (Fig. 2a, for example, ERK5-IN-1 or GW869810X) and values near zero correspond to unselective compounds (for example, GSK1269851A; Supplementary Table 3). CATDS analysis showed that KCGS comprised the largest fraction of selective inhibitors (Fig. 2b) but still contained several broad-spectrum compounds including XMD-17-51 that bound to almost 50 proteins (Supplementary Table 2). Assembling the Kinobeads data into a compound–target selectivity matrix revealed several clusters of selective inhibitors for a specific kinase (for example, epidermal growth factor receptor (EGFR); Extended Data Fig. 4a and Supplementary Table 3).

Next, we mined the data set for new potential chemical probes. These must meet certain criteria in terms of selectivity and target engagement^4^. For the Kinobeads data, these criteria were translated as follows: affinity (${{{K}}}_{{\rm{d}}}^{{\rm{app}}}$ < 1 µM) and CATDS_most potent_ > 0.5. An additional and important aspect is to account for targets outside the kinase space. For instance, ERK5-IN-1 is a selective compound in the Kinobead assay. However, the reported pharmacology in cells is mainly due to inhibition of BRD4 that cannot be detected by Kinobeads^24^. Because a full assessment of all potential targets of a compound is not possible, we limit the scope of any compound mentioned below to that of a ‘probe candidate’.

About 330 kinase inhibitors fulfilled these criteria and they target 72 kinases from all kinase subfamilies (Fig. 2c, Extended Data Fig. 4b and Supplementary Table 3). At the time of writing, the portal chemicalprobes.org (ref. ^25^) listed 123 compounds targeting 133 human protein kinases (April 2022) of which 74 compounds targeting 89 kinases were endorsed for use as specific and selective modulator of the respective target. Our data contribute compounds for an additional 43 kinases. The current work also identified probe candidates for 29 kinases that already had such designated molecules (Fig. 2c blue circles). The compound set investigated by Kinobeads here, contained 11 previous chemical probes. Only one (GSK583) also fulfilled our criteria and one other (CCT24474) barely missed the chosen threshold (CATDS_CHECK1_ 0.46). For four compounds (CCT251545, FM-381, GSK481, NVS-PAK1-1), the designated target could not be enriched by Kinobeads or it was not expressed in the cell lines used. A further four compounds (GNF-5, THZ1 and THZ531, NVP-2) failed to reveal their targets. This may be due to slow binding of the covalent inhibitors THZ1 (ref. ^26^) and (presumably) THZ531. For allosteric binder GNF-5, failure to detect BCR-ABL competition is not clear. The Kinobeads technology typically only scores ATP-competitive inhibitors unless allosteric compounds alter the ATP pocket conformation in a way that makes it inaccessible for the affinity resin (for example, observed for the AKT inhibitor MK2206, ref. ^3^). However, alternative displacement assays using bioluminescence resonance energy transfer (BRET) and time-resolved fluorescence energy transfer as readouts have detected the interaction^27,28^. For NVP-2, it is also not clear why CDK9 was not detected because many other compounds were. Because all identified off-targets did not pass the affinity threshold, an assessment of selectivity is not possible from the results obtained here. WZ4003 had poor selectivity in the Kinobeads assay and should, therefore, only be used with caution and including appropriate controls.

The Kinobead profiling data revealed probe candidates for well-studied kinases as well as understudied kinases. For this assessment, a target-centric version of CATDS can be used, which is calculated in an analogous fashion as the score of the most potent target (‘CATDS score’ section). The largest number of candidates (37 compounds) were detected for the well-studied kinase EGFR, reflecting the strong medicinal chemistry efforts over the years for this important target (Extended Data Fig. 4c). We also found selective compounds for the clinically relevant drug targets MET and FLT3 (Extended Data Fig. 4d,e), including RO0272148-000 as a potent (${{{K}}}_{{\rm{d}}}^{{\rm{app}}}$ = 99 nM) and selective (CADTS_FLT3_ = 0.87) FLT3 inhibitor. We note that even though several FLT3 inhibitors are approved drugs, these are not good tools for basic research on FLT3 as they often target many other proteins. The only off-target of RO0272148-000 in the Kinobead assay was ACOX1 (${{{K}}}_{{\rm{d}}}^{{\rm{app}}}$ = 1,158 nM), the first enzyme of the fatty acid beta-oxidation pathway. To what extent this off-target binding is biologically relevant, remains to be investigated. We also discovered several selective EPHB6 binders. EPHB6 is a pseudokinase and modulates cell adhesion and migration when stimulated by ephrin-B2 (ref. ^29^). It is unclear how ATP-competitive small molecules would modulate nonenzymatic functions of pseudokinases but with selective molecules such as GW459057A (${{{K}}}_{{\rm{d}}}^{{\rm{app}}}$ = 162 nM) in hand, this biology may be further explored (Extended Data Fig. 4f). In addition, the molecule may serve as a warhead for the development of proteolysis targeting chimeras against EPHB6. This is interesting as pseudokinases are gaining interest in drug discovery owing to their physiological roles associated with various human diseases^30^.

### GSK986310C is a potent and selective SYK inhibitor

**Fig. 3:**
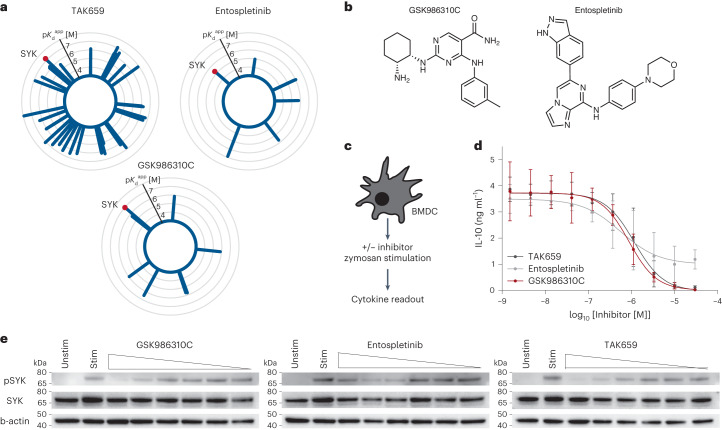
GSK986310C is a probe candidate for SYK. **a**, Radar plots depicting the targets and binding affinities of the tool compound GSK986310C and the two clinical SYK inhibitors entospletinib and TAK659. Each spike represents one target and its length corresponds to the affinity of the compound–protein interaction. **b**, Chemical structures of GSK986310C and entospletinib. **c**, Schematic representation of the cytokine secretion assay in primary murine BMDCs. **d**, Enzyme-linked immunosorbent assays showing dose-dependent reduction of IL-10 levels in response to GSK986310C, entospletinib and TAK659. Data are presented as mean values ± s.e.m. of *n* = 3 biological replicates. **e**, Western blot readout of phospho-SYK (Tyr525/525) in BMDCs treated with inhibitors after 30 min of Zymosan stimulation. One out of three biological replicates is shown. Source data

The spleen tyrosine kinase (SYK) has been validated as a target for the treatment of a number of hematological cancers, autoimmune disorders and other inflammatory conditions^31,32^. Several SYK inhibitors (for example, fostamatinib, entospletinib and TAK659) are approved drugs or under evaluation in clinical trials^33^. According to previous Kinobeads profiling results, fostamatinib, its active metabolite and TAK659 bound many other proteins beside SYK (Fig. 3a and published data^3^ and Supplementary Table 2). Hence, these SYK inhibitors are of insufficient quality to function as chemical probes to study the cellular function of the protein. By contrast, entospletinib had fewer targets but SYK was not the most potently inhibited protein (Fig. 3a,b and Supplementary Table 2).

**Extended Data Fig. 5:**
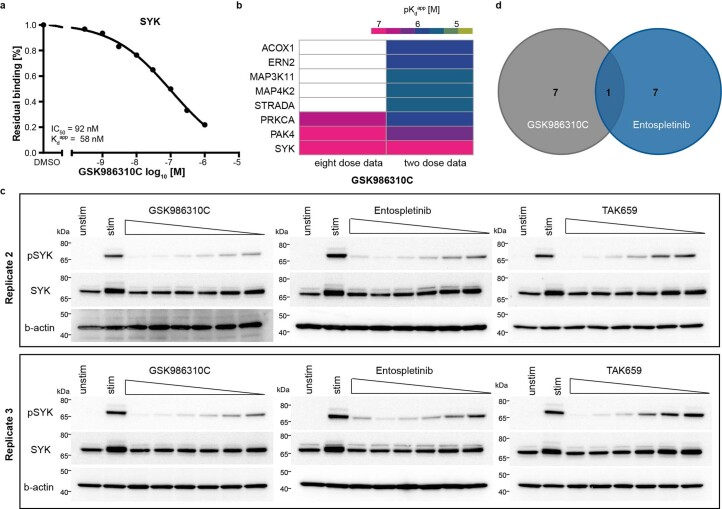
Selective SYK inhibitors. **a**, Dose response curve of the Kinobeads competition experiment with GSK986310C for SYK. **b**, Heat map representing the target space of GSK986310C obtained by two dose and eight dose Kinobeads assay. Color code reflects the affinity of the compound:protein interaction. **c**, Western blot readout of phospho-SYK (Tyr525/525) in BMDCs treated with inhibitors after 30 min of Zymosan stimulation. Here biological replicate 2 and 3 are shown. **d**, Venn diagram showing the overlap of GSK986310C and Entospletinib targets. SYK is the only shared target of the two inhibitors.

Mining the Kinobeads data identified GSK986310C as a rather selective and potent SYK binder (CATDS_SYK_ = 0.51; ${{{K}}}_{{\rm{d}}}^{{\rm{app}}}$ = 80 nM; Fig. 3a,b). A subsequent full dose–response experiment confirmed potent and selective binding to SYK (${{{K}}}_{{\rm{d}}}^{{\rm{app}}}$ = 58 nM, CATDS_SYK_ = 0.60; Extended Data Fig. 5a,b). Several much weaker binders (more than ten times weaker than SYK) did not confirm in the dose–response analysis indicating that low affine targets are less reproducible in the assay. To confirm that SYK binding translates into target engagement and modulation of SYK activity in cells, we subjected GSK986310C, TAK659 and entospletinib to IL-10 secretion assay using murine primary bone marrow-derived dendritic cells (BMDCs) following Zymosan stimulation. One of the effects of Zymosan is that it activates Dectin-1 signaling, in turn activating SYK and leading to increased IL-10 cytokine production (Fig. 3c)^34^. Treatment of cells with the respective SYK inhibitors before Zymosan stimulation indeed led to decreased levels of IL-10 in a dose-dependent fashion (Fig. 3d). To show that SYK target engagement in cells is responsible for this observation, we determined the phosphorylation status of SYK-Tyr525/526 within the ATP-binding pocket. In line with the cytokine readout, we observed a dose depended reduction of pTyr525/526 indicating SYK inhibition in live cells (Fig. 3e and Extended Data Fig. 5c). The above data show that GSK986310C and entospletinib are SYK inhibitors in cells and we argue that they should be used alongside each other as a chemical probe set to ascertain that any effects these molecules may have on cells, can indeed be attributed to the inhibition of SYK (Extended Data Fig. 5d).

### Selective inhibitors for CK2

**Fig. 4:**
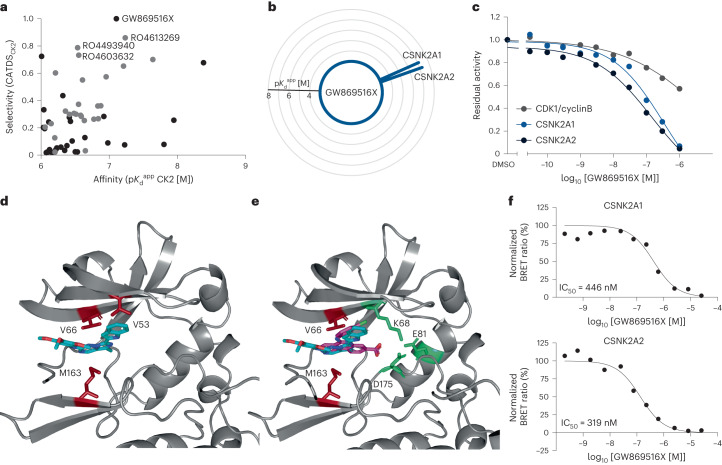
Selective and potent CK2 inhibitors. **a**, Scatter plot of selectivity (CATDS_CK2_) and affinity (${{{\rm{p}{\it{K}}}}}_{{\rm{d}}}^{{\rm{app}}}$) of CK2 inhibitors. Compounds of the quinolinyl-methylene-thiazolinone chemotype are marked in gray. **b**, Radar plot depicting the target space and binding affinities of GW869516X. **c**, Recombinant kinase activity assay of GW869516X for CDK1/cyclinB, CSNK2A1 and CSNK2A2. **d**, Crystal structure of the ATP pocket of CK2α bound to GW869516X (PDB 7ZWE, blue). The central fused ring interacts with the back of the ATP site and the hinge region, stacking between Met163 and Val66 that are marked in red. **e**, Crystal structure of the ATP pocket of CK2α with GW869516X (PDB 7ZWE, blue) and CX-4945 (PDB 3PE1, purple) superimposed. The highly conserved residues Lys68, Asp175 and Glu81 within the ATP site are highlighted in green. **f**, Dose-dependent in-cell target engagement assays for CSNK2A1 and CSNK2A2 (NanoBRET in HEK293T cells) for GW869516X. One out of two biological replicates are shown.

**Extended Data Fig. 6:**
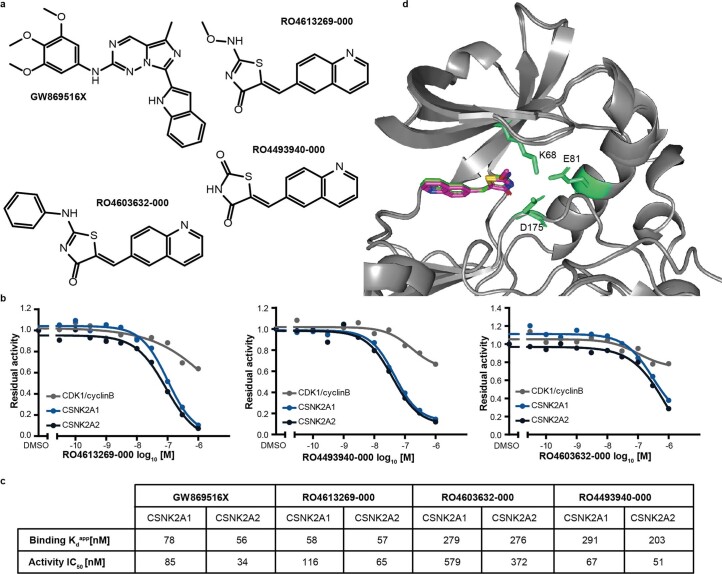
Selective CK2 inhibitors. **a**, Chemical structure of CK2 inhibitors. **b**, Kinase activity assays of the indicated compounds for CDK1/cyclinB, CSNK2A1 and CSNK2A2 validated the binding results obtained by Kinobeads. **c**, *K*_d_^app^ and IC_50_ values for CSNK2A1 and CSNK2A2 in Kinobeads screen (binding) and in recombinant kinase assays (activity). **d**, The highly conserved residues within the ATP site are highlighted (green) on the structure of RO4613269-000 (PDB:7A4Q; purple) bound to CK2α with RO4493940-000 (PDB:7ZWG; green) superimposed.

CK2 is involved in the regulation of many cellular processes including cell growth, proliferation and death. The protein is often overexpressed in cancer cells and some become addicted to CK2 activity^35,36^. As a result, CK2 has emerged as an interesting target in oncology but very few molecules (for example, CX-4945) have made it to clinical trials so far. Although CX-4945 has been reported to be potent (confirmed by Kinobeads; ${{{K}}}_{{\rm{d}}}^{{\rm{app}}}$ = 1 nM) and selective (CADTS_CK2_ = 0.89), several studies report off-target effects mediated by other proteins^3,37^. Our Kinobeads profiling data contained 64 CK2 binders some of which showed very high affinity and selectivity (Fig. 4a and Supplementary Tables 2 and 3). Among these, 25 represent the quinolinyl-methylene-thiazolinone chemotype, originally optimized for CDK1 inhibition and leading to the identification of the CDK1 inhibitor RO-3306 (ref. ^38^). From the Kinobead data, RO4613269-000, RO-4603632-000 and RO4493940-000 appeared to be selective CK2 binders (CATDS_CK2_ of 0.86, 0.73, 0.70) with nanomolar affinity (${{{K}}}_{{\rm{d}}}^{{\rm{app}}}$ of 58, 279 and 291 nM, respectively, Fig. 4a and Extended Data Fig. 6a). Since members of this chemotype had not been comprehensively profiled for kinases before, it was not known that compounds of this chemotype can bind CK2. Inhibition of CK2 activity by all three compounds was confirmed using recombinant activity assays that showed very good correspondence with the Kinobeads binding data (Extended Data Fig. 6b,c and Supplementary Table 4). None of the three compounds showed potent inhibition of CDK1 binding or activity, which agrees with published data^39^.

Another potent and selective inhibitor for CK2 was GW869516X (CATDS_CK2_ of 1; ${{{K}}}_{{\rm{d}}}^{{\rm{app}}}$ of 78 nM; IC_50_ of 85 nM) representing an imidazotriazine chemotype unrelated to the RO-compound series (Fig. 4b,c, Extended Data Fig. 6a,c and Supplementary Tables 2–4). This was a surprising finding as a previous study reported more than 70% inhibition of binding of 33 kinases in a broad KINOMEscan assay (covering 468 kinases) for this compound. Only CSNK2A1 and CSNK2A2 were confirmed as targets using Kinobeads, 20 kinases showed no inhibition of binding and 11 kinases were not detected in the Kinobeads assay (Supplementary Table 4). The reasons for this discrepancy are currently not clear.

To investigate the structural basis for the observed selectivity of GW869516X (according to Kinobeads), we determined the crystal structure of CK2α in complex with GW869516X (Protein Data Bank (PDB) 7ZWE). The structure clearly shows GW869516X binding in the ATP pocket of CK2α (Fig. 4d and Supplementary Table 4). The central fused ring interacts with the back of the ATP site and the hinge region, stacking between Met163 and Val66. The indole group sits underneath Val53, projecting away from the pocket. The trimethoxybenzene group protrudes from the ATP site interacting with the end of the hinge region and the top of the αD loop. Examination of the structure of ATP site of a number of representative kinases indicated that a number of residues that cluster around Lys68 are highly conserved across a large number of kinases, whereas residues in the hinge region and around the top of the αD loop are less conserved^40^. GW869516X does not interact with the highly conserved Lys68 or its surrounding residues (Fig. 4e). Comparison with the binding mode of CX-4945 (PDB 3PE1)^41^ highlighted the differences in the selectivity of the two compounds (Fig. 4e). As mentioned, CX-4945 has been shown in a number of studies to potently inhibit a large number of other kinases^37^. Indeed, the binding mode of CX-4945 to seven of these kinases has been determined crystallographically^42–44^. CX-4945 binds to all these kinases in a highly conserved binding mode that is dominated by its interaction with the conserved residue equivalent to Lys68 and this is likely the factor that drives its promiscuity. Likewise, when the binding modes of RO4493940-000 and RO4613269-000 (PDB 7ZWG and 7A4Q) were determined by crystallography in the current study (Extended Data Fig. 6d), they showed that the thiazole head group interacts with the conserved residue Lys68. These compounds, similar to CX-4945, showed inhibition of a few other kinases that is likely, in part, because of the interaction with Lys68. Of note, RO4493940-000 contains a rhodanine-like substructure and rhodanine is among a list of pan assay interference compounds (PAINS)^45^. However, the Kinobeads data do not indicate that RO4493940-000 is a promiscuous binder or that CK2 is a false positive hit.

CK2 target engagement by GW869516X in cells, was confirmed by NanoBRET assays resulting in IC_50_ values of 446 and 319 nM for CK2A1 and CK2A2, respectively (Fig. 4f and Supplementary Table 4). GW869516X was less potent in NanoBRET versus Kinobead assays (Extended Data Fig. 6c) likely because cells contain far higher ATP concentrations than lysates and the plasma membrane of the cell also constitutes a physical barrier.

### GSK902056A and GSK949675A are probe candidates for PKN3

The serine/threonine kinase PKN3 is an understudied kinase whose molecular function and downstream targets are largely unknown^46^. In recent studies, PKN3 has been functionally linked to metastasis, invasion and tumor growth making it a potential target for drug discovery in oncolgy^47,48^. A liposomal small interfering RNA (siRNA) (Atu027) against PKN3 is currently under investigation in clinical trials for solid tumors and pancreatic cancer^49,50^. In 2019, a covalent PKN3 inhibitor (JZ128) that targets Cys840 in PKN3 was reported^51^. JZ128 was developed based on the structure of THZ1, a CDK7 inhibitor with reported off-target binding to PKN3. In addition, a small, focused library of 4-anilinoquin(az)olines PKN3 inhibitors with cellular activity was reported. However, the described compounds had off-targets within the kinome and in-cell target engagement was rather low (1.3 µM) even for the most potent compound (UNC-CA94)^52^. Hence, there is a clear need for further selective and potent PKN3 inhibitors.

**Fig. 5:**
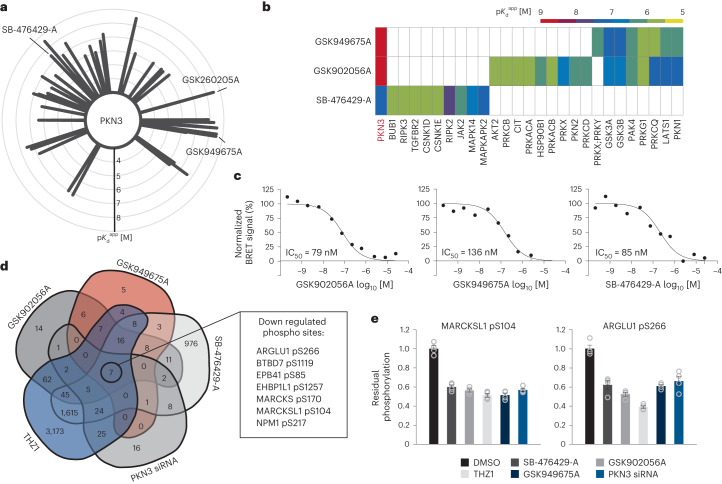
Compounds targeting PKN3. **a**, Radar plot depicting compounds that bind PKN3 with a particular affinity. Each spike represents one compound and the length indicates the affinity of the interaction. **b**, Heat map illustrating the targets and affinities of three selected PKN3 inhibitors. **c**, Dose-dependent in-cell PKN3 target engagement assays (NanoBRET in HEK293T cells) for the same compounds as in **b**. One out of three biological replicates is shown. **d**, Venn diagram showing the number of regulated phosphorylation sites identified in RKO cells in response to 1 h treatment and 1 µM of the respective compounds or 48 h of siRNA-mediated PKN3 knockdown. Commonly regulated phosphorylation sites are shown in the text box. **e**, Examples for potential PKN3 substrates and their response to the conditions shown in **d**. Data are presented as mean values ± s.e.m. of *n* = 4 biological replicates.

**Extended Data Fig. 7:**
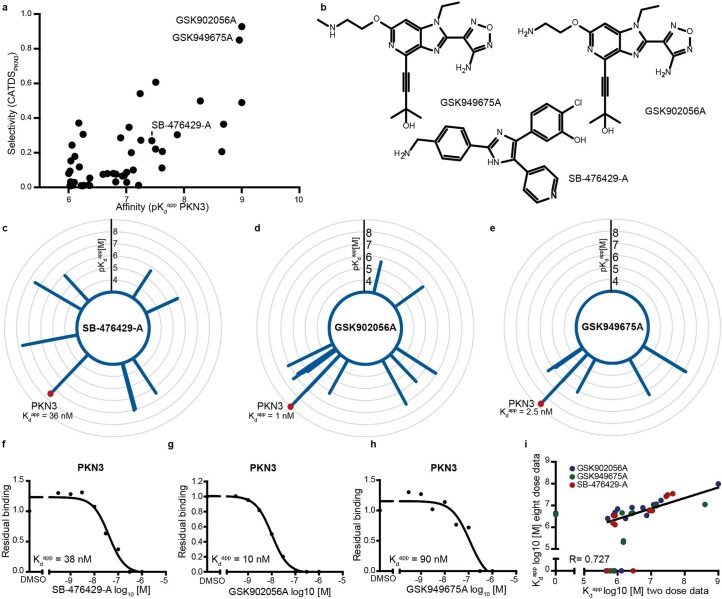
Compounds targeting PKN3. **a**, Selectivity of PKN3 inhibitors as obtained by the PKN3-specific CATDS_PKN3_ score. Inhibitors in the top right corner are the most potent and most selective PKN3 inhibitors within the screened panel. **b**, Chemical structure of three tool compounds that show binding to PKN3. **c-e**, Radarplots depicting the target space and binding affinities of the three PKN3 inhibitors (**c**) SB-476429-A, (**d**) GSK902056A and (**e**) GSK949675A. **f-h**, Full dose response curve of Kinobeads competition experiment with (**f**) SB-476429-A, (**g**) GSK902056A and (**h**) GSK949675A for PKN3. **i**, Correlation of *K*_d_^app^ values obtained by two dose and eight dose Kinobeads pulldown experiment.

**Extended Data Fig. 8:**
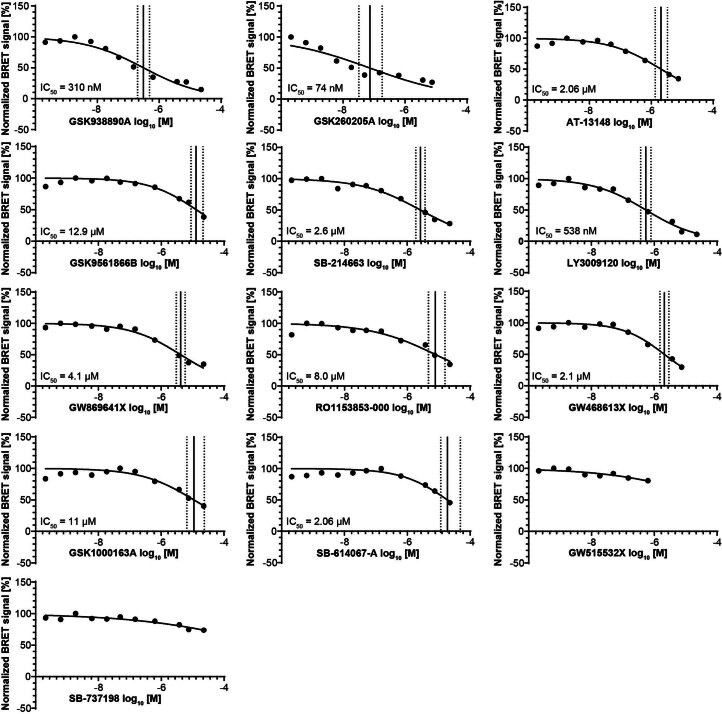
Cellular target engagement of PKN3 inhibitors. Dose dependent reduction of the BRET signal with increasing inhibitor concentrations were observed for eleven compounds indicating PKN3 target engagement in cells. One out of four biological replicates is shown.

The Kinobeads data identified 49 PKN3 binders of which GSK902056A (CATDS_PKN3_ of 0.93; ${{{K}}}_{{\rm{d}}}^{{\rm{app}}}$ of 1 nM) and GSK949675A (CATDS_PKN3_ of 0.86; ${{{K}}}_{{\rm{d}}}^{{\rm{app}}}$ of 2.5 nM) stood out in terms of potency and selectivity (Fig. 5a and Extended Data Fig. 7). SB-476429-A is less selective but a useful control compound as it only shares PKN3 as a common target (CATDS_PKN3_ of 0.21; ${{{K}}}_{{\rm{d}}}^{{\rm{app}}}$ of 36 nM; Fig. 5b and Extended Data Fig. 7). The two-dose Kinobeads data were confirmed by full dose–response measurements (${{{K}}}_{{\rm{d}}}^{{\rm{app}}}$ of 10, 90 and 38 nM for GSK902056A, GSK949675A and SB-476429-A, respectively; Extended Data Fig. 7f–h and Supplementary Table 5) and the two measurements were well correlated (Pearson’s *r* = 0.716, Extended Data Fig. 7i). Cellular target engagement of PKN3 binders was assessed by NanoBRET assays^28^ and 14 of the 16 chosen compounds showed a dose-dependent reduction of the normalized BRET signal (Fig. 5c, Extended Data Fig. 8 and Supplementary Table 5). Again, compounds were less potent in the NanoBRET than in the Kinobeads assay. Still, GSK902056A potently engaged PKN3 in cells with an IC_50_ of 79 nM, GSK949675A with 136 nM and SB-476429-A with 85 nM and all three compounds showed higher levels of in-cell target engagement and a higher selectivity window than the reported compounds mentioned above^52^. Taken together, GSK902056A may be the current best in class PKN3 probe and may be a good starting point for further optimization to develop more potent PKN3 inhibitors in the future.

**Extended Data Fig. 9:**
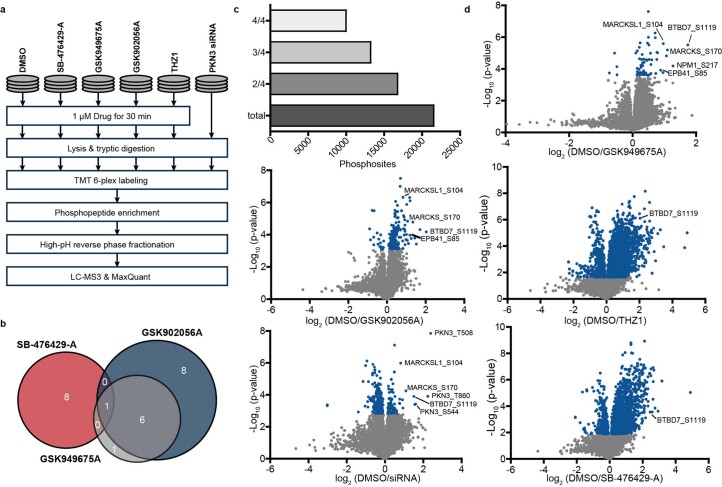
Phosphoproteome analysis of RKO cells in response to PKN3 inhibitors. **a**, Schematic representation of the phosphoproteomic workflow (performed in four biological replicates). **b**, Venn diagram showing the overlap of the targets of three PKN3 inhibitors. PKN3 is the only shared target of the inhibitors. **c**, Number of identified phospho sites in four biological replicates. **d**, Volcano plots showing the extent and statistical significance of regulated phospho sites by four PKN3 inhibitors and PKN3 siRNA. Statistically significantly regulated phospho sites are marked in blue (two sided T-test, FDR = 0.01; s0 see material and methods).

**Extended Data Fig. 10:**
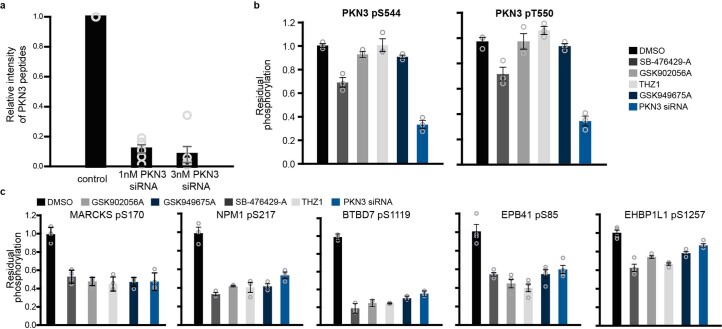
Phosphoproteome analysis of RKO cells in response to PKN3 inhibitors. **a**, Bar plot showing relative intensity of PKN3 peptides as monitored by a PRM assay. Six different peptides were monitored and sum peak areas of all fragment ions were used for analysis. Relative intensity of all six peptides were calculated and the mean value +/− s.e.m. are shown. Different siRNA concentrations were tested to knock down PKN3 in RKO cells. (**b**) Residual phosphorylation of PKN3 peptides after treatment of RKO cells with respective inhibitors or siRNA. Mean values +/− s.e.m. are shown. Error bars indicate standard deviations of *n* = 4 biological replicates are shown. (**c**) Residual phosphorylation of selected phospho sites. Error bars indicate mean values +/− s.e.m. of *n* = 4 biological replicates are displayed.

To be able to measure PKN3 activity in cells, bona fide substrates of the kinase would be needed but no such substrate has been conclusively validated yet. In search for such candidates, we compared the phosphoproteomes of RKO cells (human colorectal cancer cells expressing high PKN3 levels according to ProteomicsDB^53^) in response to the same three compounds (that only share PKN3 as a target), as well as THZ1 (a covalent inhibitor for which PKN3 is a reported off-target^51^) and in response to siRNA-mediated knockdown of PKN3 (Extended Data Fig. 9a,b). The analysis covered a total of 21,400 phosphorylation sites (p sites, Extended Data Fig. 9c and Supplementary Table 5) and showed that THZ1 and SB-476429-perturbed a large number of p sites, reflecting the engagement of many target kinases in cells. The respective numbers were far smaller for GSK949675A, GSK902056A (all four drugs used at 1 µM for 1 hour) or the siRNA experiment (48 h of knockdown; Fig. 5d, Extended Data Fig. 9d and Supplementary Table 5). Successful PKN3 knockdown (89 and 92% for 1 and 3 nM siRNA, respectively) was confirmed by parallel reaction monitoring (a quantitative mass spectrometry-based protein expression assay; Extended Data Fig. 10a). The reported activation loop p sites of PKN3 (S544 and T550) showed a marked reduction following PKN3 knockdown and a substantial reduction by SB-476429-A, but not by the other compounds (Extended Data Fig. 10b). Because the binding modes of GSK949675A, GSK902056A and SB-476429 are not known, the lack of A-loop phosphorylation reduction by the GSK compounds does not mean that PKN3 is not inhibited by the compounds. In line, THZ1 covalently binds to a Cys residue remote from the ATP pocket of PKN3 and which renders PKN3 inactive but it did not lead to a change in A-loop phosphorylation.

Seven p sites on seven different proteins were consistently down-regulated in all replicates (*n* = 4) and treatment conditions making these the strongest candidates for direct or indirect targets of PKN3 in RKO cells (Fig. 5d,e and Extended Data Fig. 10c). The reduction of pS104 on MARCKSL1 validated the approach because this site had previously been identified as a potential downstream target of PKN3 (ref. ^51^). In addition, six of the seven candidates were found to be PKN3 substrates in a very recent large-scale investigation of kinase–substrate relationships^54^, three of which with extremely high confidence (greater than the 97th percentile). From this study, we constructed a PKN3 substrate motif and four of the seven candidates contain the central Ser-Phe residues making these the best candidates for direct PKN3 substrates (Supplementary Table 5). Clearly, further experiments are required to confirm PKN3 as the kinase responsible for these particular phosphorylation sites.

## Discussion

In this study, we have generated a large resource of kinase–drug interactions on the basis of nearly 1,200 compounds from medicinal chemistry efforts and the Kinobead chemical proteomics approach. The body of 500,000 drug–protein interactions covers about 250 protein and lipid kinases and is available for further mining in ProteomicsDB^53,55^ and we highlight a number of cases how this resource may be used. The analysis promotes about 350 potential probe candidates that can be further investigated and identified 40 kinases for which no chemical probes had been available yet.

While this chemical proteomics screen was overall very successful, it is not without its shortcomings. The Kinobead assay does not cover all kinases; hence it is possible, that the designated target of a compound cannot be identified. It is also possible that a chemical probe candidates is less (or more) selective than suggested by our data because Kinobeads assess target engagement in lysates not in cells. It is possible, that the cellular target engagement and selectivity of probe candidates is higher or lower than expected from the Kinobead profiles. The former may result from ATP concentrations being much higher in cells than in lysates or for targets that gain their cellular activity by forming protein complexes in cells (for example, CDKs, PI3Ks). The latter can arise from, for example, poor compound penetration or the activity of efflux pumps. We also note that the assay was not optimized for differences in binding kinetics of individual compound. The apparent interaction constants reported in this study may, therefore, not necessarily be accurate for slow binders such as histone deacetylase inhibitors (as a result of slow on-rates) or certain covalent compounds such as THZ1 (as a result of slow reaction). This can be easily addressed by performing Kinobead assays using different drug preincubation times but doing so was not feasible for more than 1,000 compounds. Similarly, systematic phosphoproteome profiling of all compounds may be one way to address cellular selectivity in more detail, but performing experiments on such a scale is not yet experimentally tractable.

It should be noted that more than half of the human kinome is still without chemical probes. In part, this is due to the limited set of kinases covered by the Kinobead assay but it is also very possible that the chemical space able to address human kinases is not yet comprehensive. Together with the fact that no compounds have been found for targeting many human kinases with a clear disease connotation warrant and require substantial further chemical biology and chemical proteomics efforts in the future. In light of recent advances in computational structural biology^56^ we think that the data and online resource provided in this study add substantially to this important field of research.

## Methods

### Affinity matrix and compounds

PKIS and PKIS2 and KCGS were obtained from the Structural Genomics Consortium (SGC). The KCGS library can be requested here: https://www.sgc-unc.org/request-kcgs (February 2023). The Roche library was provided by Hoffmann-La Roche AG (Basel) and is not commercially available. Clinical kinase inhibitors were purchased from commercial sources (Selleckchem, MedChemExpress, Active Biochem or LC Laboratories). Kinase inhibitor affinity matrices (Kinobeads ε) were prepared in house as previously described^14^.

### Cell lines and lysis

K-562 (chronic myeloid leukemia), COLO-205 (colon cancer) and MV-4-11 (acute monocytic leukemia) cells were cultured in Roswell Park Memorial Institute 1640 medium (Biochrom). SK-N-BE(2) (neuroblastoma) cells were grown in DMEM/Ham’s F-12 (1:1) and OVCAR-8 (ovarian cancer) cells were cultured in Iscove’s modified Dulbecco’s medium (Biochrom GmbH). All were supplemented with 10% (v/v) fetal bovine serum (Biochrom GmbH). Cell lines were tested internally for mycoplasma contamination. Cells were lysed in 0.8% IGEPAL (octylphenoxypolyethoxyethanol), 50 mM Tris-HCl pH 7.5, 5% glycerol, 1.5 mM MgCl_2_, 150 mM NaCl, 1 mM NA_3_VO_4_, 25 mM NaF, 1 mM dithiothreitol (DTT) and supplemented with protease inhibitors (SigmaFast, Sigma) and phosphatase inhibitors (PI-III; in house, composition resembling phosphatase inhibitor cocktail 1, 2 and 3 from Sigma-Aldrich). Protein concentration was determined by Bradford assay and aliquots were stored at −80 °C.

### Kinase inhibitor profiling with Kinobeads (Kinobeads pulldowns)

Kinobeads pulldown experiments were performed as described previously^13,14,17^ with minor modification. Briefly, the cell lysate mixture used for inhibitor profiling was generated from COLO-205, K-562, MV-4-11, SK-N-BE(2) and OVCAR-8 lysates by mixing them in a 1:1:1:1:1 ratio. The protein concentration was adjusted to 5 mg ml^−1^. Kinase inhibitors were spiked into 0.5 ml of cell lysate at final concentrations of 100 and 1,000 nM (or DMSO, 0.3, 1, 3, 10, 30, 100, 300 and 1,000 nM for full dose experiments). In addition, six DMSO control pulldowns and three lestaurtinib pulldown experiments as control were distributed over a 96-well plate. Lysates were incubated for 45 min at 4 °C. Subsequently, lysates were incubated with Kinobeads ε (17.5 µl of settled beads) for 30 min at 4 °C. To assess the degree of protein depletion from the lysates by Kinobeads, the flow through of the DMSO control was recovered for a pulldown of pulldown experiment where the lysate was incubated a second time with fresh Kinobeads. Beads were washed in three steps with buffer containing 0.4%, 0.2% and no IGEPAL. Bound proteins were reduced with 50 mM DTT in 8 M Urea, 40 mM Tris-HCl pH 7.4 for 30 min at room temperature. After alkylation with 55 mM chloroacetamide, the urea concentration was reduced to 1–2 M and proteins were digested with trypsin. Acidified peptides were desalted and concentrated using SepPak tC18 µEluation plates (Waters). Samples were frozen, dried by vacuum centrifugation and stored at −20 °C.

### LC–MS/MS of Kinobeads pulldowns

Nano liquid chromatography–electrospray ionization with mass spectrometry (LC–ESI–MS) measurements of two-dose and full dose Kinobeads pulldown samples were performed using a Dionex Ultimate3000 nano high-performance liquid chromatography (HPLC) coupled online to an Orbitrap HF (Thermo Fisher Scientific) mass spectrometer. Peptides were delivered to a trap column (100 µm × 2 cm, packed in house with Reprosil-Pur C18-GOLD, 5 µm resin, Dr. Maisch) and washed for 10 min with 0.1% formic acid at a flow rate of 5 µl min^−1^. Peptide separation was performed on an analytical column (75 µm ID × 40 cm packed in house with Reprosil-Pur C18, 3 µm resin, Dr. Maisch) at a flow rate of 300 nl min^−1^ using a 52 min gradient ranging from 5 to 33% solvent B (0.1% formic acid, 5% DMSO in acetonitrile (ACN)) in solvent A (0.1% formic acid in 5% DMSO). The Orbitrap HF was operated in data-dependent acquisition and positive ionization mode. MS1 spectra were acquired in the Orbitrap over a mass-to-charge (*m*/*z*) range of 360–1,300 *m*/*z* at a resolution of 120,000 (60,000 resolution for full dose Kinobeads pulldown samples) using an automatic gain control (AGC) target value of 3 × 10^6^ charges or a maximum injection time of 10 ms. Up to five (12 for eight dose pulldowns) peptide precursors were selected for fragmentation by higher energy collision-induced dissociation (HCD) using 25% normalized collision energy (NCE), an isolation width of 1.7 *m*/*z*, a maximum injection time of 22 ms (75 ms for eight dose pulldowns) and an AGC values of 1 × 10^5^ charges (2 × 10^5^ for full dose pulldowns). Resulted fragment ions were recorded in the Orbitrap with a resolution of 15 K. A previous experimentally obtained inclusion list containing approximately 3,700 kinase peptide *m*/*z* and their corresponding retention time values was enabled. Dynamic exclusion was set to 30 s.

### Peptide and protein identification and quantification of Kinobeads pulldowns

Raw data files were searched with MaxQuant software (v.1.6.0.1) using standard settings unless otherwise described^18^. Tandem mass spectra were searched against all canonical protein sequences as annotated in the UniProt reference database (human proteins only, 20,230 entries, downloaded v.06.07.2017). Carbamidomethylated cysteine was set as fixed modification. Variable modifications included phosphorylation of serine, threonine or tyrosine, oxidation of methionine and N-terminal protein acetylation. Trypsin/P was specified as proteolytic enzyme with up to two missed cleavage sites. Label-free quantification and match between runs were enabled. Results were filtered for 1% peptide and protein false discovery rate (FDR) using a target-decoy approach using reversed protein sequences.

### Data analysis of Kinobeads pulldowns

Each kinase inhibitor was processed together with all DMSO controls of the same plate. Additionally, each search was supplemented with five high quality DMSO controls for consistent peptide identification and protein grouping. The resultant file (proteinGroups.txt) was used for subsequent filtering, normalization, data visualization and target annotation that was automatically performed by a data processing pipeline built in house that used R (including the ‘drc’ and ‘heatmap’ packages). First, reverse hits, potential contaminants and not quantified proteins in the DMSO control samples were discarded. Protein raw and label-free quantification intensities were normalized to the median DMSO control intensity to obtain relative residual binding intensities for each protein group at every inhibitor concentration and standard deviations of the DMSO control intensity were calculated. IC_50_ values were estimated based on the following equation

$${\rm{IC}}_{50}=\left[I\right]\times \frac{100-{{\mathrm{inhibition}}}}{{{\mathrm{inhibition}}}}$$

where [*I*] is the inhibitor concentration that was used for IC_50_ calculation and ‘inhibition’ the relative residual binding intensity. The formula was applied to the inhibitor concentration that showed residual binding close to 0.5. Estimated ${{{K}}}_{{\rm{d}}}^{{\rm{app}}}$ values were then calculated by multiplying the estimated IC_50_ values with a protein-dependent correction factor that was limited to a maximum value of one. The correction factor accounts for the depletion of a target protein from the lysate by Kinobeads. The depletion can be measured by the ratio of the amount of protein captured in two consecutive pulldowns of the same DMSO control lysate^57^. In this study, correction factors were determined in separate experiments using the same lysate mixture and the same bead batch. Correction factors were set to the median of all correction factors. Targets of kinase inhibitors were annotated using the previously published random forest classifier^19^. A training data set was annotated manually. Hereby, a protein was considered as target if the relative residual binding intensity was reduced by at least 30% at the highest compound concentration and if the standard deviation of the DMSO intensity was substantially lower than the overall reduction of the median relative intensity. Additionally, the number of unique peptides and MS/MS spectra were included as target selection criteria. Protein intensity in DMSO controls was also taken into account. Targets were considered as direct Kinobeads binders if annotated in Uniprot.org as protein kinase, lipid kinase, nucleotide binder, helicase, ATPase and GTPase as well as FAD and heme containing proteins. Most other target proteins were interaction partners and/or adaptor proteins of kinases, and were termed indirect Kinobeads binders. Binding affinities were reported as ${\rm{p}}{{K}}_{{\rm{d}}}^{{\rm{app}}}$ values, which is the negative logarithm of ${{{K}}}_{{\rm{d}}}^{{\rm{app}}}$ in mol l^−1^.

For the target classifier, the targets of a limited number of compounds were annotated manually as described above and used as training set for the random forest model. The trained classifier assigns a target probability score to each quantified proteins in the data set ranging from 0 to 100%. Proteins with a target probability higher than 93.5% were annotated as target of the compound of interest and proteins below the threshold were annotated as no targets. The cutoff was used to reach the minimum overall number of false positive and false negative hits.

Full dose Kinobeads pulldowns were analyzed as reported previously^3,14^.

### CATDS score

The CATDS is a measure of the target engagement of a specific protein at a certain drug concentration relative to the target engagement of all targets at that drug concentration. It was calculated as reported previously^3^ and as shown in the following equation

$${\mathrm{CATDS}}_{\mathrm{target}}=\frac{{\sum ({{\mathrm{target}}\; {\mathrm{engagement}}})}_{{{\mathrm{target}}\; {\mathrm{of}}\; {\mathrm{interest}}}}}{{\sum ({\mathrm{target}}\; {\mathrm{engagement}})}_{{\mathrm{all}}\; {\mathrm{targets}}}}$$

To determine the target engagement of a protein at any concentration, a slope of one was assumed and the top was set to one and the bottom was set to zero to generate a dose–response curve based on the two-dose data. For curve fitting a four-parameter log-logistic regression model was used.

$${I}_{\mathrm{rel}}\left(c\right)=b+\frac{t-b}{1+{\mathrm{e}}^{s\times (\log \left(c\right)-\log \left(i\right))}}$$

where *c* is the compound concentration and the four free parameters are the plateau of the fit *b* (bottom), the maximal residual binding *t* (top) and the hill slope *s* of the curve at the infection point *I* (half-maximum effective concentration). The CATDS_target_ was determined at the respective ${{{K}}}_{{\rm{d}}}^{{\rm{app}}}$ concentration of the targeted protein for each inhibitor.

### Kinase activity assay

Kinase activity assays were performed by ProQinase. Dose-dependent activity inhibition of CDK1/cyclinB, CK2alpha1 and CK2alpha2 were measured using a FlashPlateTM-based radiometric assay at *K*_M_ (ATP) of the respective kinase.

### Cytokine secretion assay and phospho-SYK readout in response to SYK inhibitors

Primary BMDCs were obtained from 9-week-old C57BL/6 mice that were maintained under standard specific pathogen-free conditions. BMDCs were differentiated for 7 days in Roswell Park Memorial Institute medium (Gibco) and granulocyte-macrophage colony-stimulating factor. On day seven, BMDCs were seeded in 96-well plates at 10^5^ cells per well in 100 µl of culture medium followed by incubation for 4 h at 37 °C. Subsequently, cells were incubated with SYK inhibitors for 30 min followed by stimulation with Zymosan (final concentration of 50 µg ml^−1^; Invivogen) and dispersed in culture medium for 24 h. Western blotting was performed using 15 μg of cell lysate and the following antibodies phospho-SYK-Tyr525/526 (1:1,000; Cell Signaling, catalog no. 2710), total SYK (1:1,000; Cell Signaling, catalog no. 2712) and b-actin (1:1,000; Proteintech, catalog no. 66009-1). For cytokine secretion quantification, cell culture medium was collected and concentrations of IL-10 in the supernatant were determined using mouse enzyme-linked immunosorbent assay kits (IL-10: Invitrogen) according to the manufacturer’s protocol. Experiments were performed in biological triplicates.

### X-ray crystallography

Expression and purification of CK2α was done as described before^37^. Crystallization, soaking of ligands and structure determination was done as described previously. X-ray diffraction data were collected at the Diamond Light Source on the i24 and iO4 beamline and data from automated data processing with autoProc were used for the structure determination. All coordinated have been deposited to the PDB under accession numbers 7ZWE, 7A4Q and 7ZWG. Data collection and refinement statistics are shown in Supplementary Table 4.

### NanoBRET assay

The NanoBRET assay was performed as described previously^28,58^. In brief, full-length PKN3, CSNK2A1 or CSNK2A2 ORF (PKN3 vector was a kind gift of Promega, Vectors for CSNK1A1 and CSNK1A2: NV2981, NV1191) cloned in frame with a C-terminal NanoLuc-fusion, respectively, were transfected into HEK293T cells using FuGENE HD (Promega, E2312) and proteins were allowed to express for 20 h. Serially diluted inhibitor and NanoBRET Kinase Tracer K5 (Promega, N2530) at 500 nM (PKN3) or NanoBRET Kinase Tracer K10 (Promega, N2840) at 500 nM (CSNK1A1 and CSNK1A2) were pipetted into 384-well plates using an Echo acoustic dispenser (Labcyte). The PKN3, CSNK2A1 or CSNK2A2 transfected cells were added at a density of 2 × 10^5^ cells per ml after trypsinization and resuspending in Opti-MEM without phenol red (Life Technologies). The system was allowed to equilibrate for 2 h at 37 °C and 5% CO_2_ before BRET measurements. BRET signaling was measured by adding NanoBRET NanoGlo Substrate and Extracellular NanoLuc Inhibitor (Promega) according to the manufacturer’s protocol. Filtered luminescence was measured on a PHERAstar plate reader (BMG Labtech) equipped with a luminescence filter pair (450 nm BP filter (donor) and 610 LP filter (acceptor)). Competitive displacement data were then graphed using GraphPad Prism software (v.5.01) using a three-parameter curve fit with the following equation

$$Y={\mathrm{bottom}}+\frac{({\mathrm{top}}-{\mathrm{bottom}})}{1+{10}^{\left({\mathrm{log}}{\mathrm{IC}}_{50}-X\right)\times {\mathrm{hillslope}}}}$$

Experiments were performed with at least two biological and two technical replicates.

### PKN3 siRNA knockdown

RKO cells were cultured in Iscove’s modified Dulbecco’s medium (Biochrom) supplemented with 10% (v/v) fetal bovine serum. Knockdown of PKN3 in RKO cells was performed by siRNA (siPOOL2 targeting human PKN3, NCBI gene ID 29941; siPOOLs Biotch) according to the instructions from the manufacturer. Briefly, PKN3 siRNA was diluted with Opti-MEM to a concentration of 0.05 μM. siRNA dilution was then mixed in a 1:1 ratio with Lipofectamine RNAiMAX (diluted by a factor 100 in Opti-MEM; Thermo Fisher Scientific) by vortexing and incubated for 5 min at room temperature. The transfection mixture was transferred to the bottom of a fresh 10 cm cell culture plate and RKO cells were added in a density of 1 × 10^6^ cells per ml. Knockdown was controlled by parallel reaction monitoring assay after 48 h of incubation. Therefore, cells were washed twice with phosphate-buffered saline and lysed by scraping in the presence of 100 µl of lysis buffer (0.8% IGEPAL, 50 mM Tris-HCl pH 7.5, 5% glycerol, 1.5 mM MgCl_2_, 150 mM NaCl, 1 mM NA_3_VO_4_, 25 mM NaF, 1 mM DTT, protease inhibitors (SigmaFast, Sigma) and phosphatase inhibitors). Lysates were centrifuged and proteins were alkylated with chloroacetamide (55 mM) and run into a 4–12% NuPAGE gel (Invitrogen, approximately 1 cm). In-gel digestion was performed according to standard procedures.

Based on previous Kinobeads LC with tandem mass spectrometry (LC–MS/MS) runs various PKN3 peptides were selected to generate an inclusion list with 10 min monitoring windows. In addition, Prosit was used to generate a spectral library. Nanoflow LC–ESI–MS/MS measurements were performed with a DionexUltimate 3000UHPLC+ system coupled to a Q Exactive HF mass spectrometer (Thermo Fisher Scientific). After reconstitution in 0.1% FA, peptides were delivered to a trap column (75 mm × 2 cm, packed in house with 5 mmC18 resin; Reprosil-Pur AQ, Dr. Maisch) and washed using 0.1% formic acid at a flow rate of 5 ml min for 10 min. Subsequently, peptides were transferred to an analytical column (75 mm × 45 cm, packed in house with 3 mm C18 resin; Reprosil Gold, Dr. Maisch) applying a flow rate of 300 nl min^−1^ and separated using a 30 min linear gradient from 5 to 35% LC solvent B (0.1% FA, 5% DMSO in ACN) in LC solvent A (0.1% FA in 5% DMSO). The mass spectrometer was operated in positive ionization mode. Full scan MS1 spectra were recorded in the Orbitrap mass analyzer from 150 to 2,000 *m*/*z* at a resolution of 15,000 (at *m*/*z* 200) using an AGC target value of 3 × 10^6^ and a maximum injection time of 100 ms. For targeted MS2 scans, the scheduled precursors were isolated (isolation window 0.7 *m*/*z*) and fragmented via HCD using a NCE of 25%. MS2 spectra were recorded in the Orbitrap mass analyzer at a resolution of 15,000 using an AGC target value of 2 × 10^5^ and a maximum injection time of 100 ms.

The generated .raw files were imported into Skyline for data filtering and analysis. Confident peptide identification was carried out based on matching to the predicted library and the dotp. Peaks were integrated using the automatic peak finding function followed by the manual curation of all peak boundaries and transitions. The summed area under the fragment ion traces for every transition was exported. All fragment ion traces were summed up for one peptide and relative intensities to the control samples were calculated. To determine the knockdown efficiency, relative intensities of six peptides were taken into account. A complete knockdown was observed after 48 h and a final siRNA concentration of 1 nM.

### Drug and siRNA-perturbed phosphoproteome analysis

For global phosphoproteomic analysis of PKN3 inhibitors, RKO cells were treated with 1 µM GSK949675A, THZ1, GSK902056A, SB-476429A or DMSO for 1 h in four biological replicates or PKN3 were knocked down as described. After treatment, cells were washed twice with PBS and lyzed by adding 300 µl of lysis buffer (40 mM Tris-HCl pH 7.6, 8 M Urea, EDTA-free protease inhibitor complete mini and phosphatase inhibitor cocktail). Lysates were sonicated (ten cycles, 30 s on, 30 s pause, at 4 °C) and subsequently cleared by centrifugation for 20 min at 21,000*g* at 4 °C. Protein concentration was determined by Bradford assay and 300 µg protein per condition was digested. After reduction with 10 mM DTT and alkylation of cysteine residues with 50 mM chloroacetamide, the Urea concentration was reduced to 1.5 M by adding six volumes of 40 mM Tris/HCl pH 7.6. Trypsin was added to a protease-to-protein ratio of 1:50 and digestion was performed over night at 37 °C and 700 r.p.m. on a thermoshaker. Samples were cooled down to room temperature and acidified to a pH < 3 with 0.5% trifluoroacetic acid (TFA) and desalted using 50 mg SepPak columns (Waters; wash solvent 0.07% TFA in deionized water; elution solvent 0.07% TFA, 50% ACN). Subsequently, samples were frozen at −80 °C and dried by vacuum centrifugation. Peptide concentrations were determined by NanoDrop 2000 spectrophotometer and peptide amounts were adjusted. Dried peptides were labeled with TMT6plex as published previously^59^. One tandem mass tag (TMT) channel was used for each drug treatment (126 = SB-476429-A, 127 = GSK902056A, 128 = THZ1, 129 = GSK949675A, 130 = DMSO, 131 = PKN3 siRNA). Phosphopeptides were enriched using column based Fe-IMAC as described previously^60^. Subsequently, phosphopeptides were separated into six fractions using high pH reversed-phase stage tips as described before^61^. Samples were dried by vacuum centrifugation.

Nanoflow LC–MS/MS measurement of TMT-labeled phosphopeptides was performed using a Dionex Ultimate3000 nano HPLC coupled online to an Orbitrap Fusion Lumos Tribride (Thermo Fisher Scientific) mass spectrometer. Peptides were delivered to a trap column (75 μm × 2 cm, packed in house with 5 μm C18 resin; Reprosil-Pur AQ, Dr. Maisch) and washed for 10 min with 0.1% FA at a flow rate of 5 μl min^−1^. Subsequently, peptides were transferred to an analytical column (75 μm × 45 cm, packed in house with 3 μm C18 resin; Reprosil Gold, Dr. Maisch) at 300 nl min^−1^ and separated within a 90 min gradient ranging from 4 to 32% solvent B (0.1% FA, 5% DMSO in ACN) in solvent A (0.1% FA in 5% DMSO). MS1 spectra were recorded in the Orbitrap from 360 to 1,300 *m*/*z* at a resolution of 60,000 using an AGC target value of 4 × 10^5^ charges and a maximum injection time of 20 ms. MS2 spectra were recorded in the Orbitrap at 15,000 resolution after HCD fragmentation using 35% NCE, an AGC target value of 5 × 10^4^, maximum injection time of 22 ms and an isolation width of 0.7 *m*/*z*. The first mass was fixed to 100 *m*/*z*. The number of MS2 spectra was limited by a top ten method. For TMT quantification, an additional MS3 spectrum was acquired in the Orbitrap over a scan range of 100–1,000 *m*/*z* at 15,000 resolution (AGC of 1 × 10^5^, maximum injection time of 50 ms). For this, fragment ions were selected by multi-notch isolation in the Quadrupole, allowing a maximum of ten notches and subsequently fragmentation by HCD at 55% NCE. Dynamic exclusion was set to 90 s.

Peptide and protein identification and quantification were performed using MaxQuant with its built in search engine Andromeda. Tandem mass spectra were searched against all canonical protein sequences as annotated in the UniProt reference database (human proteins only, 20,230 entries, downloaded 6 July 2017). Carbamidomethylated cysteine was set as fixed modification. Variable modifications included phosphorylation of serine, threonine or tyrosine, oxidation of methionine and N-terminal protein acetylation. Trypsin/P was specified as proteolytic enzyme with up to two missed cleavage sites. TMT6plex reporter ions were specified for quantification and isotope impurities of TMT batches were specified in the configuration of modifications to allow automated correction of TMT intensities. Results were filtered for 1% peptide and protein FDR using a target-decoy approach using reversed protein sequences.

All four replicates were searched together. Decoy and potential contaminants were removed. Within one replicate the total sum of each TMT channel was calculated and normalized to the DMSO control (total sum normalization). Additionally, the average intensity for each phosphopeptide per replicate was normalized to the average intensity of the same phosphopeptide across all replicates (row wise normalization). The Perseus software (v.4.1.31.9) was used for Student’s *t*-tests (two sided) using log-transformed TMT intensities. Statistical tests were corrected for multiple testing by an FDR of 1%. S0 was computed for each statistical test separately in R (function ‘samr’). Only phosphopeptides that were detected in at least three of four replicates were considered for analysis. GraphPad and excel were used for data visualization.

### Reporting summary

Further information on research design is available in the Nature Portfolio Reporting Summary linked to this article.

## Online content

Any methods, additional references, Nature Portfolio reporting summaries, source data, extended data, supplementary information, acknowledgements, peer review information; details of author contributions and competing interests; and statements of data and code availability are available at 10.1038/s41589-023-01459-3.

### Supplementary information

Reporting Summary

Supplementary Table 1 Compound annotation.

Supplementary Table 2 Drug matrix of target affinity values.

Supplementary Table 3 Selectivities of tool compounds.

Supplementary Table 4 Results related to CK2 follow-up experiments.

Supplementary Table 5 Results related to PKN3 follow-up experiments.

### Source data

Source Data Fig. 3 Unprocessed western blots.

Source Data Extended Data Fig. 4 Unprocessed western blots.

## Data Availability

The proteomic data, including the UniProt reference database, are available at the ProteomeXchange Consortium (http://proteomecentral.proteomexchange.org) via the MassIVE partner repositories with the data set identifier MSV000092248, as well as at ProteomicsDB (www.proteomicsdb.org). Crystal structure coordinates and structure factors have been deposited to PDB under accession numbers 7ZWE, 7A4Q and 7ZWG. Source data are provided with this paper.
