# Supplementary material for: Chemical proteomics reveals the target landscape of 1,000 kinase inhibitors
Source: Nat Chem Biol. 2023 Oct 30;20(5):577–85. doi: 10.1038/s41589-023-01459-3 (PMC11062922; doi:10.1038/s41589-023-01459-3)
Supplement: Supplementary file 1 — Reporting Summary [file 41589_2023_1459_MOESM1_ESM.pdf]

nature research

Corresponding author(s): Bernhard Kuster

Last updated by author(s): Sep 8, 2023

Reporting Summary

Nature Research wishes to improve the reproducibility of the work that we publish. This form provides structure for consistency and transparency in reporting. For further information on Nature Research policies, see our Editorial Policies and the Editorial Policy Checklist.

Statistics

For all statistical analyses, confirm that the following items are present in the figure legend, table legend, main text, or Methods section.

n/a	Confirmed	
☐	☒	The exact sample size (n) for each experimental group/condition, given as a discrete number and unit of measurement
☐	☒	A statement on whether measurements were taken from distinct samples or whether the same sample was measured repeatedly
☐	☒	The statistical test(s) used AND whether they are one- or two-sided<br>*Only common tests should be described solely by name; describe more complex techniques in the Methods section.*
☒	☐	A description of all covariates tested
☐	☒	A description of any assumptions or corrections, such as tests of normality and adjustment for multiple comparisons
☐	☒	A full description of the statistical parameters including central tendency (e.g. means) or other basic estimates (e.g. regression coefficient) AND variation (e.g. standard deviation) or associated estimates of uncertainty (e.g. confidence intervals)
☐	☒	For null hypothesis testing, the test statistic (e.g. F, t, r) with confidence intervals, effect sizes, degrees of freedom and P value noted<br>*Give P values as exact values whenever suitable.*
☒	☐	For Bayesian analysis, information on the choice of priors and Markov chain Monte Carlo settings
☒	☐	For hierarchical and complex designs, identification of the appropriate level for tests and full reporting of outcomes
☐	☒	Estimates of effect sizes (e.g. Cohen's d, Pearson's r), indicating how they were calculated

Our web collection on statistics for biologists contains articles on many of the points above.

Software and code

Policy information about availability of computer code

Data collection	Thermo Scientific Xcalibur (v 4.1.31.9) was used to collect mass spectrometry data. X-ray diffraction data were collected at the Diamond Light Source on the i24 and iO4 beamline and data from automated data processing with autoProc were used for the structure determination. Filtered luminescence was measured on a PHERAstar plate reader (BMG Labtech) equipped with a luminescence filter pair (450 nm BP filter (donor) and 610 LP filter (acceptor)).
Data analysis	MaxQuant (v 1.6.0.1) with embedded search engine Andromeda together with Uniprot reference database (v 06.09.2017) were used to analyse the proteomics experiments. Internal R script based on 'drc' and 'pheatmap' packages were further used to analyse the dose-response chemoproteomics data. Perseus (v1.6.2.3), GraphPad PRISM (v5.01) and microsoft excel (2013) were further used for data analysis.

For manuscripts utilizing custom algorithms or software that are central to the research but not yet described in published literature, software must be made available to editors and reviewers. We strongly encourage code deposition in a community repository (e.g. GitHub). See the Nature Research guidelines for submitting code & software for further information.

Data

Policy information about availability of data

All manuscripts must include a data availability statement. This statement should provide the following information, where applicable:

- Accession codes, unique identifiers, or web links for publicly available datasets
- A list of figures that have associated raw data
- A description of any restrictions on data availability

The proteomic data, including the Uniprot reference database, are available at the ProteomeXchange Consortium (http://proteomecentral.proteomexchange.org) via the MassIVE partner repository (MSV000092248), as well as at ProteomicsDB (www.proteomicsdb.org). Crystal structure coordinates and structure factors have been deposited to Protein Data Bank under accession number 7ZWE, 7A4Q and 7ZWG.

Field-specific reporting

Please select the one below that is the best fit for your research. If you are not sure, read the appropriate sections before making your selection.

☒ Life sciences ☐ Behavioural & social sciences ☐ Ecological, evolutionary & environmental sciences

For a reference copy of the document with all sections, see nature.com/documents/nr-reporting-summary-flat.pdf

Life sciences study design

All studies must disclose on these points even when the disclosure is negative.

Sample size	No sample size calculations have been performed.<br>Chemoproteomics profiling were profiled once for each drug. Cytokine secretion assay was performed in biological triplicates and technical triplicates. Activity assays for measuring IC50 values were measured by testing 10 concentrations of each inhibitor in singlicate. SYK inhibitor treatment with Immunoblot readout was measured in triplicates. Drug and siRNA-perturbed phosphoproteome analysis was performed in four biological replicates. NanoBRET assays were measured in biological triplicates.
Data exclusions	No data were excluded
Replication	Chemoproteomics profiling and activity assay constitute orthogonal techniques to validate the drugs as CK2 inhibitors and showed similar resuults. Cytokine secretion assay (performed in biological triplicates) and Immunoplot analysis of SYK phosphorylation (performed in biological triplicates) validated the drug as SYK inhibitor. NanoBRET assay (performed in biological triplicates) validated the drugs as PKN3 inhibitors. For some compounds, two dose Kinobeads data were further successfully validated with a eight dose Kinobeads pulldown experiment (singlicates).
Randomization	The order of drug profiling was random. Vehicle controls were randomly distributed over a 96-well plate.
Blinding	Blinding is not relevant to this study. All data are not subjective but based on quantitative values.

Reporting for specific materials, systems and methods

We require information from authors about some types of materials, experimental systems and methods used in many studies. Here, indicate whether each material, system or method listed is relevant to your study. If you are not sure if a list item applies to your research, read the appropriate section before selecting a response.

Materials & experimental systems

n/a	Involved in the study	
☐	☒	Antibodies
☐	☒	Eukaryotic cell lines
☒	☐	Palaeontology and archaeology
☐	☒	Animals and other organisms
☒	☐	Human research participants
☒	☐	Clinical data
☒	☐	Dual use research of concern

Methods

n/a	Involved in the study	
☒	☐	ChIP-seq
☒	☐	Flow cytometry
☒	☐	MRI-based neuroimaging

Antibodies

Antibodies used	The following antibodies were used in this study phosphoSYK Tyr525/526 (Cell signaling, #2710), total SYK (Cell signaling, #2712) and b-actin (Proteintech, #66009-1).
Validation	According to the manufacturer, Phospho-Syk (Tyr525/526) (C87C1) Rabbit mAb detects endogenous levels of Syk protein only when phosphorylated at Tyr525/526 of human Syk or Tyr519/520 of mouse Syk. It also detects Syk protein when singly phosphorylated at Tyr526 of human Syk or Tyr520 of mouse Syk. Syk Antibody detects endogenous levels of total Syk protein. It does not cross-react with other members of the Syk/Zap-70 tyrosine kinase family. B-actin antibody detects endogenous levels of total b-actin.

Eukaryotic cell lines

Policy information about cell lines

Cell line source(s)	COLO-205, MV4-11, K-562, SK-N-BE(2), OVCAR-8, RKO and HEK293T have been previously used in-house (10.1021/pr5012608, 10.1038/s41467-020-17336-9, 10.1126/science.aan4368, 10.1016/j.chembiol.2017.10.010, 10.1021/acs.jmedchem.9b01227, 10.1023/A:1013188101465) or where obtained from ATCC (American Type Culture Collection).

Authentication	Multiplex human Cell line Authentication Test (MCA) was performed by multiplexion using SNP profiling, for COLO-205, MV4-11, K-562 and SK-N-BE(2). The other cell lines were not authenticate.
Mycoplasma contamination	All cell lines were tested negative for mycoplasma contamination.
Commonly misidentified lines (See ICLAC register)	None.

Animals and other organisms

Policy information about studies involving animals; ARRIVE guidelines recommended for reporting animal research

Laboratory animals	Primary bone marrow-derived dendritic cells (BMDCs) were obtained from 9 weeks old female C57BL/6 mice which were maintained under standard specific pathogen-free conditions.<br>The room is designed in accordance with EU guideline 2010/63 as an animal housing, among other things by overpressure in the animal room, air-conditioning to an air humidity of 45-60%, a temperature of 20-24°C and light-dark rhythm of 12 hours each with twilight phase and no ultrasonic emissions.
Wild animals	No wild animals were used.
Field-collected samples	No field-collected samples were used.
Ethics oversight	All animal work was conducted in accordance with German Federal Animal Protection Laws and approved by the government of Upper Bavaria (Regierung von Oberbayern, Munich, Germany).

Note that full information on the approval of the study protocol must also be provided in the manuscript.
