# Supplementary figures and images for: Chemical proteomics reveals the target landscape of 1,000 kinase inhibitors
Source: Nat Chem Biol. 2023 Oct 30;20(5):577–85. doi: 10.1038/s41589-023-01459-3 (PMC11062922; doi:10.1038/s41589-023-01459-3)

Source Data Figur 3e

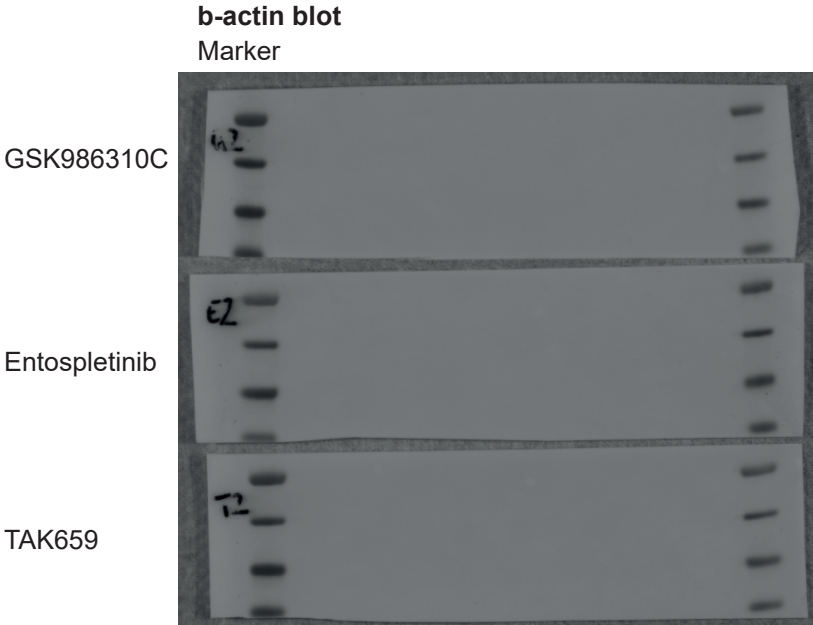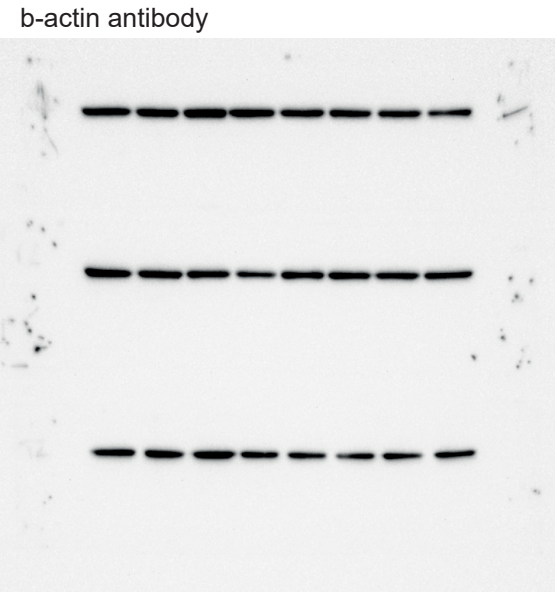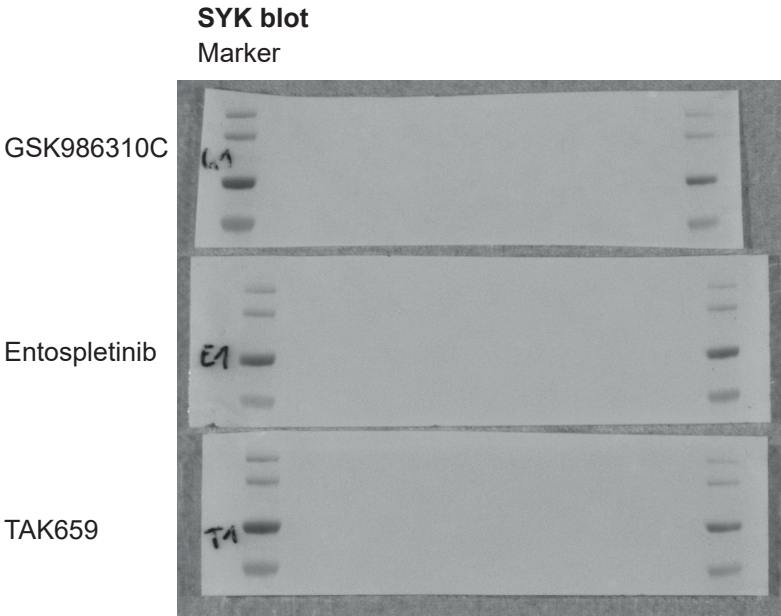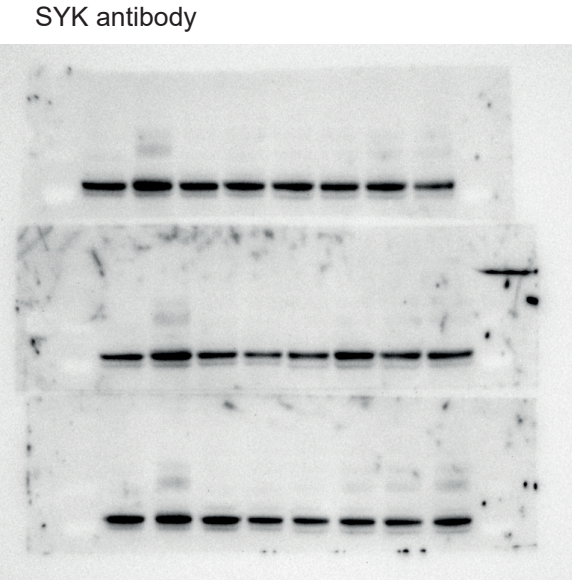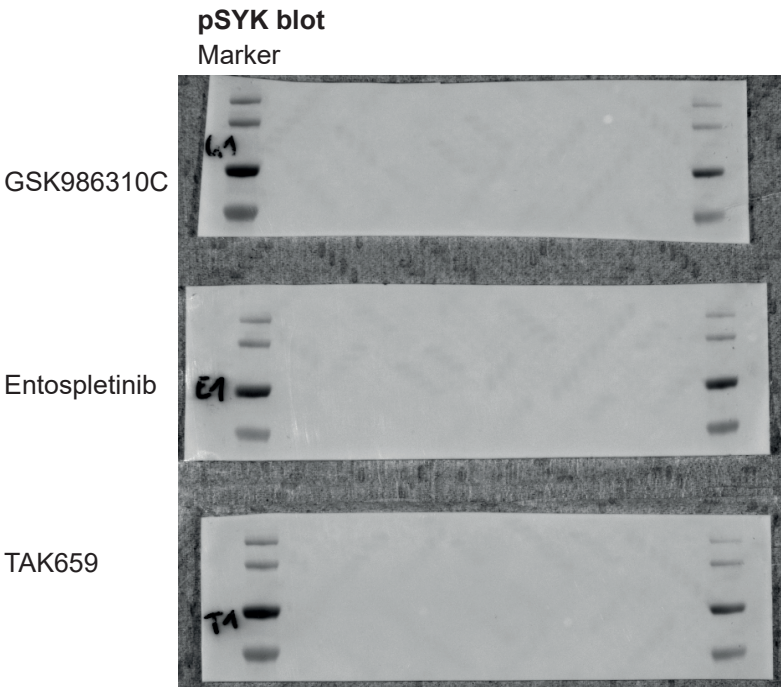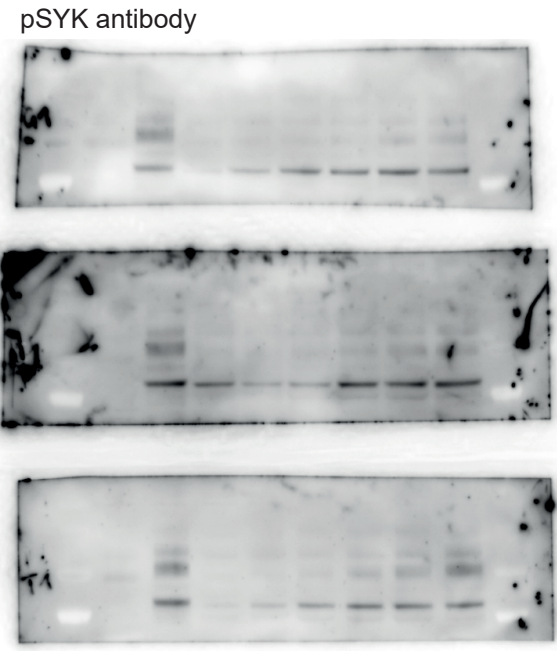

Supplement: Supplementary file 7 — Unprocessed western blots. [file 41589_2023_1459_MOESM7_ESM.pdf]
