# Supplementary material for: Chemical proteomics reveals the target landscape of 1,000 kinase inhibitors
Source: Nat Chem Biol. 2023 Oct 30;20(5):577–85. doi: 10.1038/s41589-023-01459-3 (PMC11062922; doi:10.1038/s41589-023-01459-3)

Source Data Extended Data Figur 4c

Entospletinib

b-actin

Replicate 3

Replicate 2

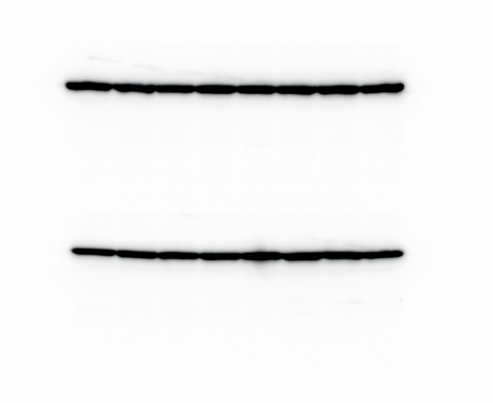

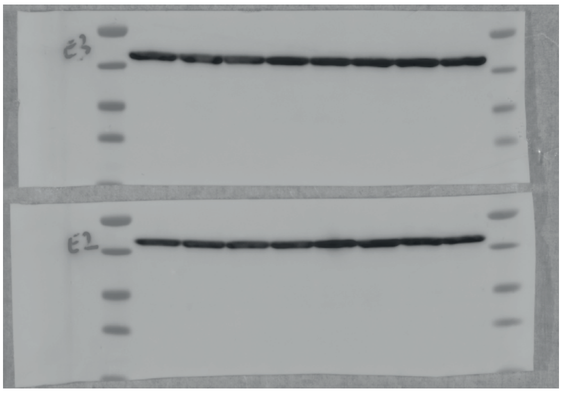

SYK

Replicate 2

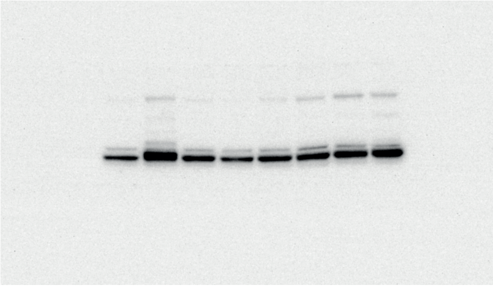

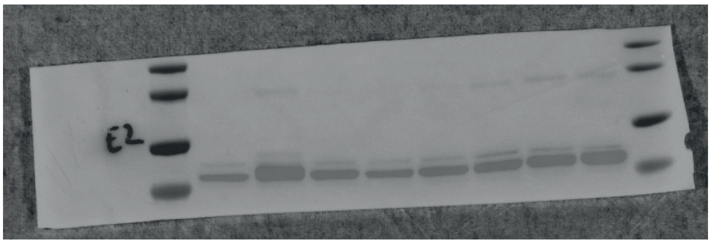

Replicate 3

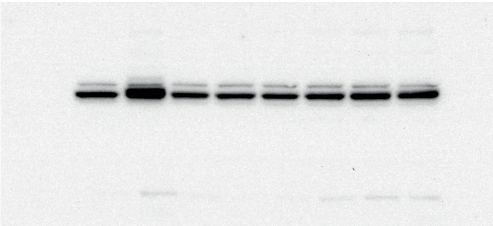

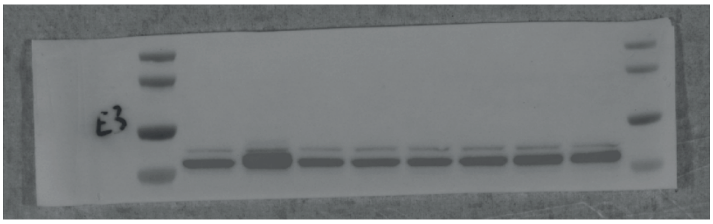

pSYK

Replicate 3

Replicate 2

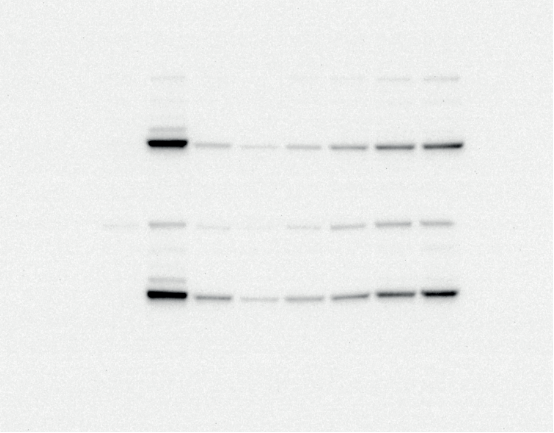

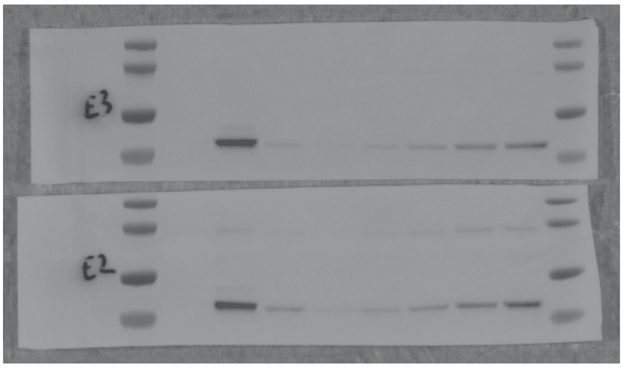

GSK986310C

b-actin

Replicate 2

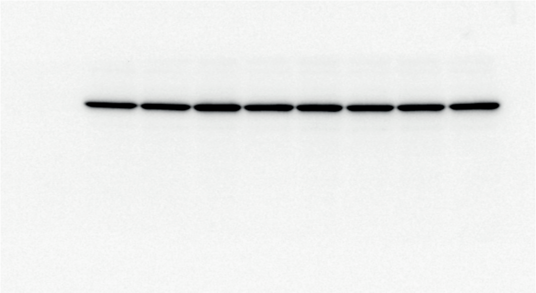

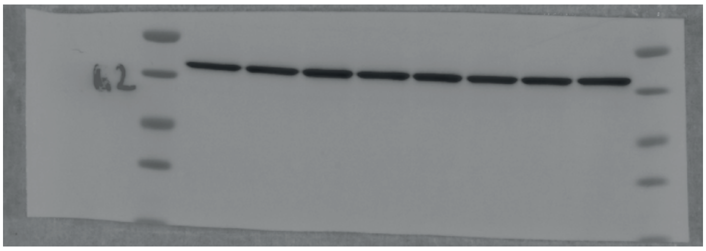

Replicate 3

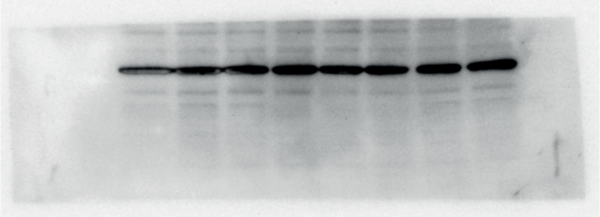

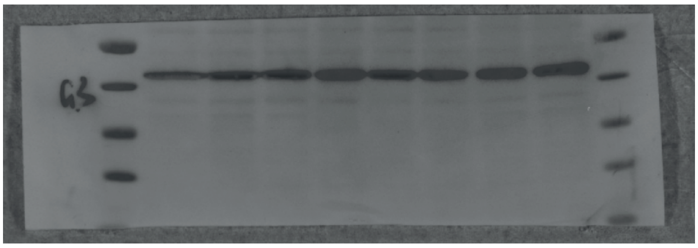

SYK

Replicate 3

Replicate 2

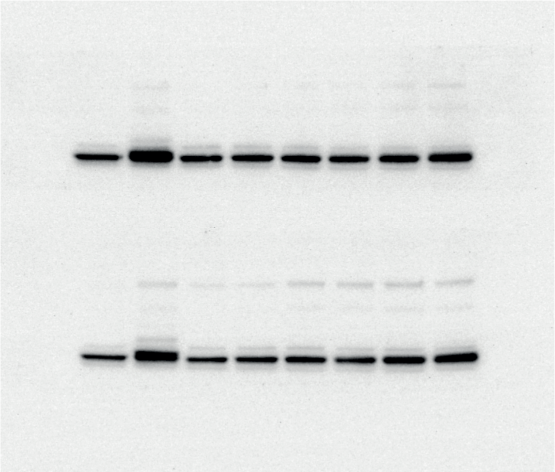

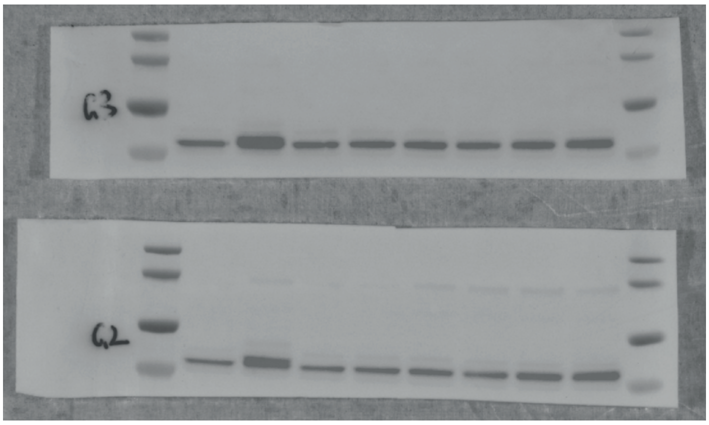

pSYK

Replicate 3

Replicate 2

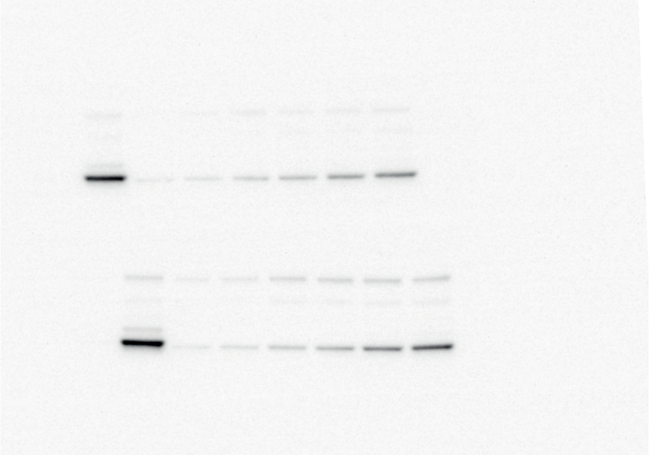

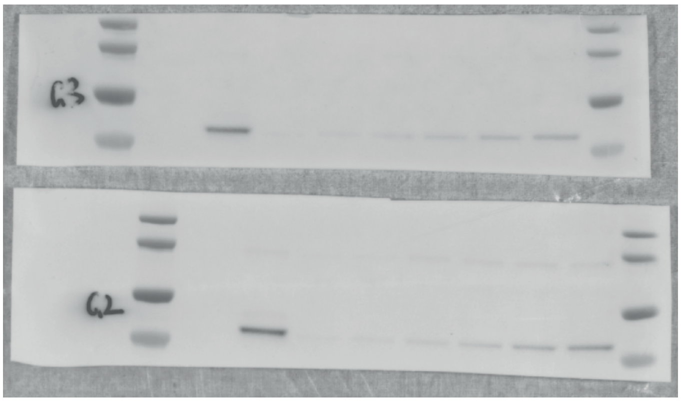

TAK659

b-actin

Replicate 3

Replicate 2

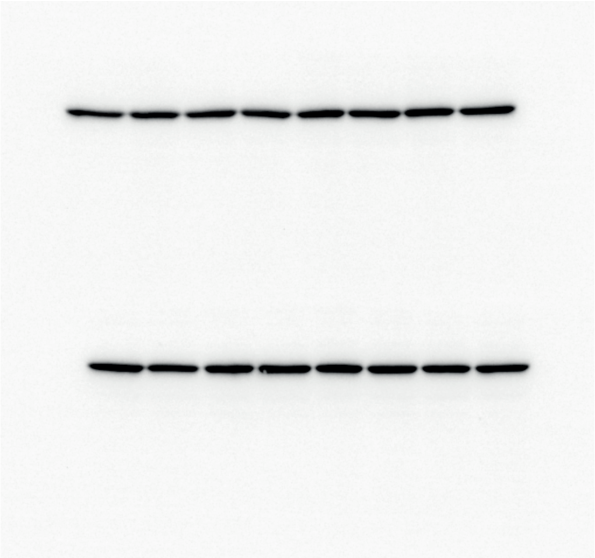

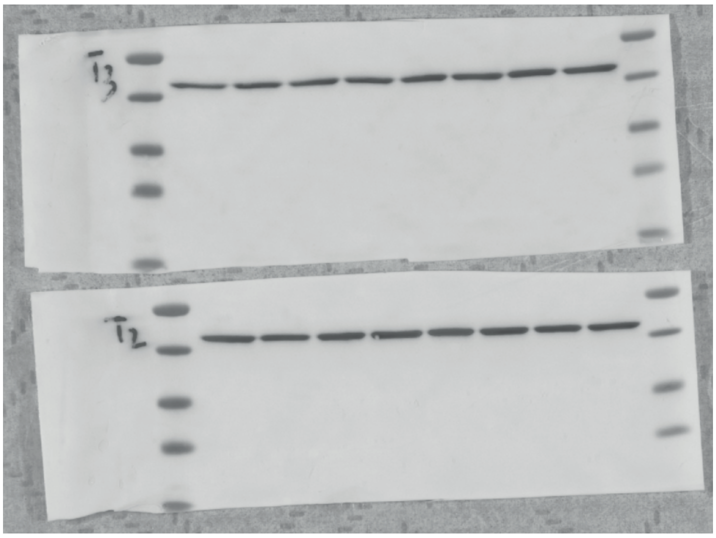

SYK

Replicate 3

Replicate 2

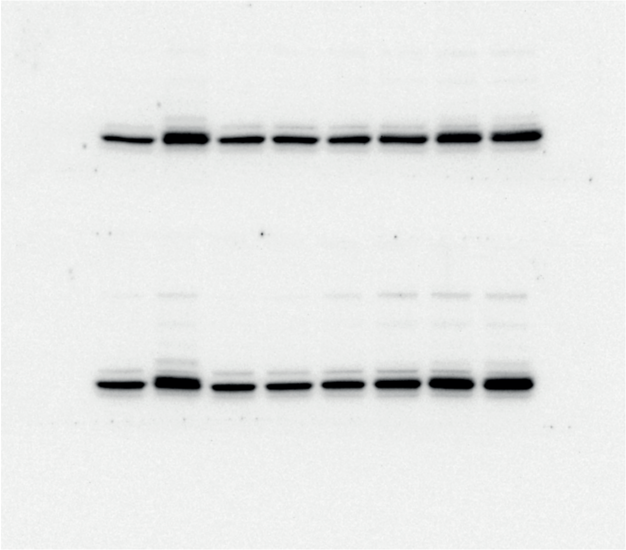

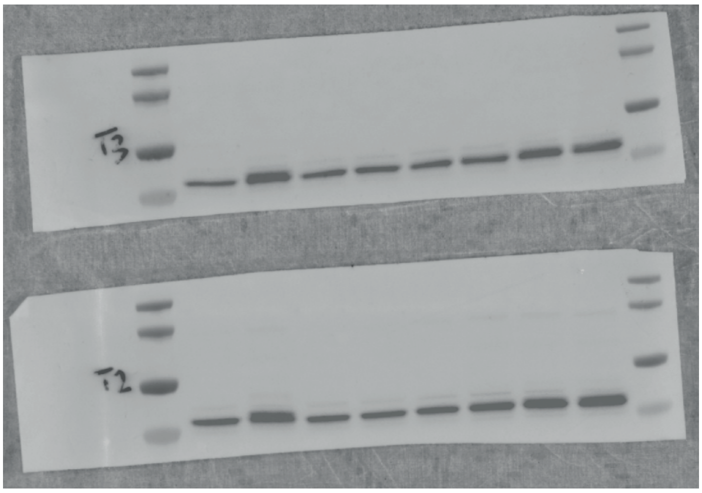

pSYK

Replicate 3

Replicate 2

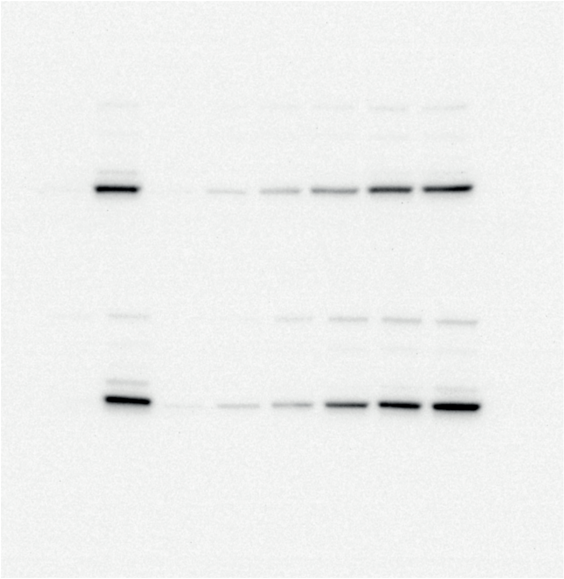

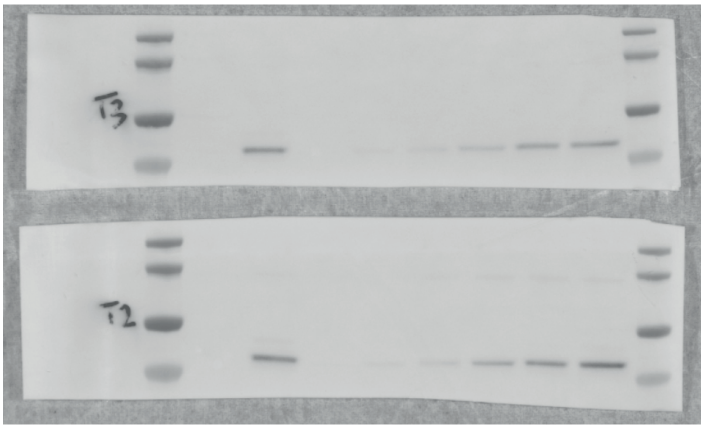

Supplement: Supplementary file 8 — Unprocessed western blots. [file 41589_2023_1459_MOESM8_ESM.pdf]
